# Phase III Prospectively Randomized Trial of Perioperative 5-FU After Curative Resection for Colon Cancer: An Intergroup Trial of the ECOG-ACRIN Cancer Research Group (E1292)

**DOI:** 10.1245/s10434-022-12705-8

**Published:** 2022-10-28

**Authors:** M. Margaret Kemeny, Fengmin Zhao, Arlene A. Forastiere, Paul Catalano, Stanley R. Hamilton, Brent W. Miedema, Nancy A. Dawson, Louis M. Weiner, Brian D. Smith, Bernard A. Mason, Stephen L. Graziano, Paul B. Gilman, Alan P. Venook, Harlan A. Pinto, Robert P. Whitehead, Peter J. O’Dwyer, Al B. Benson

**Affiliations:** 1 Icahn School of Medicine at Mount Sinai, Queens Cancer Center of NYC Health + Hospitals/Queens, Jamaica, NY USA; 2 Dana Farber Cancer Institute - ECOG-ACRIN Biostatistics Center, Boston, MA USA; 3 John Hopkins University and Sidney Kimmel Comprehensive Cancer Center, Baltimore, MD USA; 4 MD Anderson Cancer Center, Houston, TX USA; 5 University of Missouri-Ellis Fischel, Columbia, MO USA; 6 Lombardi Comprehensive Cancer Center, Washington, DC USA; 7 Wilmont Cancer Center, Rochester, NY USA; 8 Pennsylvania Hospital, Philadelphia, PA USA; 9 State University of New York Upstate Medical University, Syracuse, NY USA; 10 Lankenau Medical Center, Wynnewood, PA USA; 11 Helen Diller Family Comprehensive Cancer Center, USCF, San Francisco, CA USA; 12 Stanford Cancer Institute, Palo Alto, CA USA; 13 Nevada Cancer Institute, Las Vegas, NV USA; 14 University of Pennsylvania and Abramson Cancer Center, Philadelphia, PA USA; 15 Northwestern University, Chicago, IL USA

## Abstract

**Background:**

Studies suggest that adjuvant chemotherapy should be initiated at the earliest possible time. The Eastern Cooperative Oncology Group (ECOG) and Intergroup evaluated the effect of perioperative fluorouracil (5-FU) on overall survival (OS) for colon cancer.

**Patients and Methods:**

This phase III trial randomized patients to receive continuous infusional 5-FU for 7 days starting within 24 h after curative resection (arm A) or no perioperative 5-FU (arm B). Patients with Dukes’ B3 and C disease received adjuvant chemotherapy per standard of care. The primary endpoint of the trial was overall survival in patients with Dukes’ B3 and C disease. The secondary objective was to determine whether a week of perioperative infusion would affect survival in patients with Dukes’ B2 colon cancer with no additional chemotherapy.

**Results:**

From August 1993 to May 2000, 859 patients were enrolled and 855 randomized (arm A: 427; arm B: 428). The trial was terminated early due to slow accrual. The median follow-up is 15.4 years (0.03–20.3 years). Among patients with Dukes’ B3 and C disease, there was no statistically significant difference in OS [median 10.3 years (95% CI 8.4, 13.2) for perioperative chemotherapy and 9.3 years (95% CI 5.7, 12.3) for no perioperative therapy, one-sided log-rank *p* = 0.178, HR = 0.88 (95% CI 0.66, 1.16)] or disease-free survival (DFS). For patients with Dukes’ B2 disease, there was also no significant difference in OS (median 16.1 versus 12.9 years) or DFS. There was no difference between treatment arms in operative complications. One week of continuous infusion of 5-FU was tolerable; 18% of arm A patients experienced grade 3 or greater toxicity.

**Supplementary Information:**

The online version contains supplementary material available at 10.1245/s10434-022-12705-8.

The optimal timing of adjuvant chemotherapy after curative colon cancer surgery is unknown, but modeling of cancer dynamics implicates it as an important determinant of the therapeutic outcome.^1^ The impact of removing the primary tumor on the growth kinetics of residual tumor cells has a long history of study, and data suggest that the transition from the curable state into an incurable state could occur within a very short interval of time.^2–4^ Therefore, initiating chemotherapy at the earliest possible moment, e.g., in the immediate perioperative period, rather than at 6–8 weeks as is commonly practiced, could provide the greatest survival benefit.^5^ However, perioperative chemotherapy has been controversial because of concerns that chemotherapy may affect wound or anastomotic healing during the critical first 2 weeks after surgical tissue injury.

With these considerations in mind, the Eastern Cooperative Oncology Group (ECOG, now ECOG-ACRIN) together with the NCI Intergroup conducted a prospective randomized trial designed to test whether chemotherapy started within 24 h of surgery would be safe and more effective than standard adjuvant chemotherapy 3–6 weeks or longer after surgery. This report is the final analysis of this multi-institutional, prospective randomized trial, 15 years after the trial termination in May 2000, to study the effects of perioperative chemotherapy. The primary objective was to determine whether 1 week of infusional 5-fluorouracil (5-FU) started within 24 h of curative colon resection followed by standard 5-FU-based adjuvant chemotherapy is effective in increasing overall survival (OS) in patients with Dukes’ B3 or C (AJCC stages IIC or III) colon cancer compared with standard adjuvant chemotherapy alone. A secondary objective was to describe the treatment effect of perioperative 5-FU in patients with Dukes’ B2 colon cancer compared with no chemotherapy.

## Patients and Methods

### Patients

Patients eligible for enrollment in this study were at least 18 years old and had newly diagnosed, nonmetastatic adenocarcinoma of the colon deemed amenable to surgical cure. Patients with highly suggestive studies such as barium enemas or computed axial tomography (CAT) scans could be included without biopsies. They were able to receive chemotherapy based on adequate bone marrow reserve [WBC > 3000/mm^3^, platelets > 100,000/mm^3^], hepatic function (bilirubin < 2.0 mg/dL), and renal function (serum creatinine < 2.0 mg/dL) testing obtained within 2 weeks prior to randomization; ECOG performance status 0, 1, or 2; and the ability to give informed consent. The patients had to be randomized ≤ 2 weeks prior to colon surgery to receive either perioperative chemotherapy or no perioperative chemotherapy (step 1) and those randomized to perioperative chemotherapy had to commence treatment within 24 h of colon resection. Patients with a dual primary were ineligible, as were patients with any prior or concurrent exposure to radiation or chemotherapy for this malignant disease. The protocol was approved by the institutional review board of each participating site, and patients signed a study-specific informed consent form.

### Study Design

This prospective randomized phase III trial was conducted by the NCI Intergroup: ECOG, SWOG, NSABP, and CALGB. The study was randomized but not blinded and there was no placebo control. The trial included two steps. In step 1, patients were equally randomized to receive perioperative infusional 5-FU (arm A) or no perioperative 5-FU (arm B). Treatment assignment for patients at all institutions was obtained from the Central Randomization Desk at the ECOG Operations Office. The treatments were assigned using permutated blocks with dynamic balancing within main institutions and their affiliate networks. No stratification factors were used in randomization. If at surgery the patient’s disease was found to be metastatic, the patient was taken off protocol. All other patients randomized to arm 1 initiated perioperative chemotherapy.

**Fig. 1:**
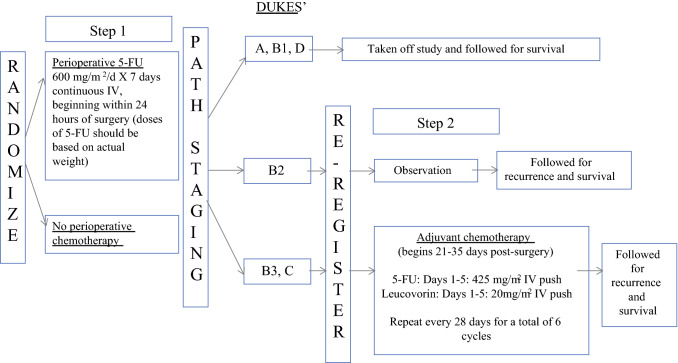
Study schema

When the surgical pathology became available, patients whose tumor stage was Dukes’ A (AJCC stage I, T1N0) or Dukes’ B1 (stage I, T2N0), were removed from the study and the chemotherapy infusion stopped. Survival data were collected. Patients whose tumor stage was Dukes’ B2 (AJCC stage IIA,T3N0 or IIB,T4aN0), Dukes’ B3 (stage IIC,T4b), or Dukes’ C (stage III), continued with their assigned treatment and were reregistered to step 2. The Dukes’ B2 patients would not receive further adjuvant chemotherapy, while the Dukes’ B3 and C patients would receive subsequent adjuvant chemotherapy per standard of care (Fig. 1, study schema).

### Treatment

Perioperative chemotherapy consisted of 5-FU administered as a continuous infusion, 600 mg/m^2^/day for 7 days starting within 24 h of surgery. The NCI Common Toxicity Criteria, version 2.0 were used for grading toxicity and adjusting doses. There were no dose modifications for perioperative continuous infusion 5-FU. WBC and platelet counts were checked on postoperative day 2 and day 5. Infusions were discontinued if any of the following occurred: leukopenia (< 3000/mm^3^), thrombocytopenia (< 100,000/mm^3^), an intervening significant postoperative complication, angina thought to be secondary to 5-FU, or grade 2 mucositis.

Adjuvant chemotherapy for patients with Dukes’ B3 and C disease consisted of either levamisole and 5-FU or leucovorin and 5-FU. From August 1993 to August 1997, levamisole and bolus 5-FU for a total of 12 months was the prescribed therapy. The dose/schedule was levamisole: 50 mg PO TID days 1–3 and 15–17; then 50 mg PO TID × 3 days beginning day 29, repeated every 14 days for 11 months and 5-FU: 450 mg/m^2^/day intravenous (IV) bolus, days 1–5, then 450 mg/m^2^ IV bolus once weekly beginning day 29 for a maximum of 11 months. Doses of 5-FU were based on actual weight. After September 1997, the adjuvant chemotherapy was changed to leucovorin and 5-FU for a total of 6 months. The dose/schedule was leucovorin: 20 mg/m^2^ IV push days 1–5 immediately followed by 5-FU 425 mg/m^2^ IV push days 1–5. A cycle of therapy consisted of five consecutive days of chemotherapy. Cycles were repeated at the end of 4 weeks (day 29), 8 weeks (day 57), and then every 4 weeks for a total of six cycles. Patients were to receive their adjuvant chemotherapy no sooner than 21 days and no longer than 35 days after the operation.

Dose modifications for adjuvant levamisole and 5-FU were as follows: (1) absolute granulocyte count (AGC) on the day of treatment ≥ 1000 and < 1500/mm^3^, 5-FU dose was omitted and treatment resumed when AGC ≥ 1500/mm^3^; for AGC < 1000/mm^3^, 5-FU was omitted and resumed when AGC ≥ 1500/mm^3^ with dose 20% dose reduction. (2) If any stomatitis or diarrhea developed, the next dose of 5-FU was omitted until complete resolution. If toxicity was CTC grade 2–4, 5-FU resumed with a 20% dose reduction. For grade 1 diarrhea or stomatitis and for hematologic toxicity, 5-FU could be re-escalated. Regardless of 5-FU dose modification, levamisole continued at full dose.

Dose modifications for adjuvant leucovorin and 5-FU were as follows: (1) for a nadir WBC 1000–2500/mm^3^ or platelets 25,000–75,000/mm^3^, 5-FU was reduced by 20% of the previous dose; for nadir WBC < 1000/mm^3^ or platelets < 25,000/mm^3^, 5-FU was reduced by 30%. (2) for WBC < 3500/mm^3^ and/or platelets < 100,000/mm^3^ at the start of a treatment cycle, 5-FU was held and counts repeated twice weekly. If the blood counts did not recover, adjuvant therapy was discontinued. (3) For grade 2 diarrhea or stomatitis, 5-FU was reduced by 20%; for grades 3–4 diarrhea or stomatitis, 5-FU was reduced by 30%; 5-FU was re-escalated only in patients experiencing grade 1 diarrhea or mucositis and for hematologic toxicity. The dose of leucovorin was not modified for any chemotherapy toxicity. Patients were followed every 3 months for 2 years, then every 6 months for 2 years and then annually.

### Statistical Analysis

The primary endpoint was to compare OS between arms A and B in patients with Dukes’ B3 and C colon cancer. The study was originally designed to detect a 9% difference in 5-year OS (60% versus 69%, 37% improvement in median OS) with 82% power, which required 800 patients with Dukes’ B3 and C colon cancer. Using data on pathologic staging, it was projected that 40% of all patients would be Dukes’ B3 or C, thus requiring a total accrual goal of 2000 patients to obtain the required 800 patients.

Accrual to this study was slower than expected, mainly because of the increasingly complex logistics of perioperative chemotherapy administration, and it was terminated early with final accrual of 314 patients with Dukes’ B3 and C disease. The study design was then revised. In the revised design, with 314 patients with Dukes’ B3 and C disease, the study had 84% power to detect a 60% improvement in median OS (5-year OS: 66% versus 77%) with one-sided type I error rate of 5% (no interim analysis had been conducted before accrual termination) using log-rank test. The revised design included one interim analysis at about 2–3 years after study closure and one final analysis at 5 years after study closure.

No formal treatment comparison was planned in the study design for patients with Dukes’ B2 disease, and there was no target accrual goal for this subgroup either. Efficacy analysis was conducted separately for patients with Dukes’ B3 and C disease and patients with Dukes’ B2 disease. Per protocol, the primary population for efficacy analyses was all eligible patients. However, because in recent years the standard for the conduct and reporting of phase III studies has shifted strongly in the direction of intent to treat (ITT) in all cases regardless of eligibility status, the primary population for all efficacy analyses in this report was all randomized patients regardless of eligibility status. Efficacy analyses in all eligible patients (per protocol) were conducted as sensitivity analysis. Analyses pertaining to adverse events (AEs) were based on all patients who received at least one dose of protocol therapy and had toxicity data. The trial was not registered, since the study was done in 1993 there was no regulation that trials be registered at that time.

OS was defined as time from randomization to death from any cause, censoring patients who had not died at the date last known alive. Disease-free survival (DFS) was a secondary endpoint, defined as time from randomization to recurrence, second invasive primary cancer, or deaths, whichever occurred first. Patients who were still alive and had no DFS events were censored at the last known follow-up disease assessment date. Patients without any follow-up data were censored at random assignment.

The distributions of DFS and OS were estimated using the Kaplan–Meier method (1958),^6^ with 95% confidence intervals calculated using Greenwood’s formula. Log-rank test and Cox proportional hazards models (1972)^7^ were used to compare DFS and OS between treatment groups. Proportional hazards assumption was examined by the Schoenfeld residuals method. Adverse events were coded and graded using the National Cancer Institute (NCI) CTC version 2.0. The incidence of treatment-related (excluding “definitely not treatment-related”) grade 3 or higher AEs (defined as number of patients experiencing the AE divided by all treated patients) were summarized for each arm using the binomial proportion and exact 95% confidence interval.

For the primary endpoint (OS in patients with Dukes’ B3 and C disease), the overall significance level was one-sided 0.05 per protocol, the nominal significance level at the final analysis was 0.048 for one-sided log rank test adjusting for the one interim analysis conducted in 2002. The significance level was set at 0.05 for two-sided tests for all other analyses.

## Results

This prospective randomized multi-institutional trial was activated on 20 August 1993 and terminated on 19 May 2000, due to slow accrual. The study stopped disease assessment for all patients in June 2005 (the expected final analysis time for the study per the revised design). Updates, however, have been continuing for survival data. This report is based on data available as of 19 February 2015. By then, the median follow-up time was 15.4 years (range: 0.03–20.3 years) for patients with Dukes’ B3 and C disease. For patients with Dukes’ B2 disease, the median follow-up was 15.8 years (range: 0.02–20.7 years).

### Patients

**Fig. 2:**
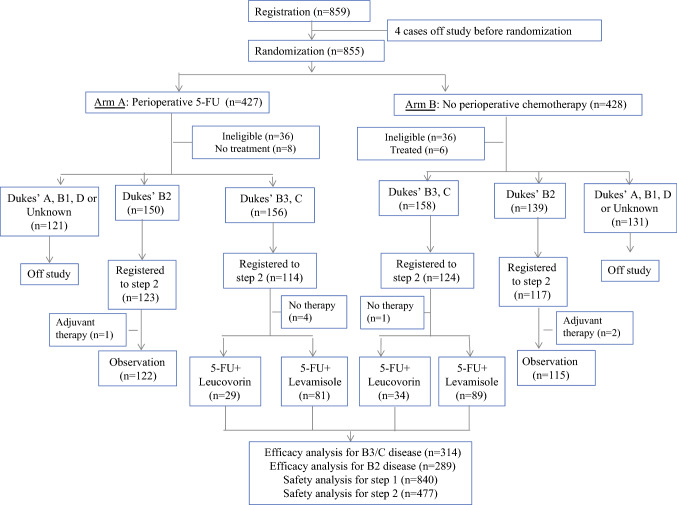
CONSORT diagram

The final accrual for the study was 859 patients. Of the 859 enrolled patients, 4 were not randomized after registration and were excluded from all analyses. A total of 855 patients were randomized (step 1): 427 to arm A (perioperative 5-FU) and 428 to arm B (no perioperative chemotherapy). Figure 2 displays the study CONSORT diagram. Seventy-two patients were ineligible for step 1, the randomization to perioperative or no perioperative 5-FU treatment, 36 in arm A and 36 in arm B. The most common reason for ineligibility was laboratory values not acquired within 2 weeks before randomization (32 patients); other reasons included rectal cancer (10 patients), dual primary (8 patients), metastatic disease (3 patients), treatment not started within 24 h of surgery (3 patients), and miscellaneous reasons in individual patients. An additional eight patients in arm A did not start their assigned treatment for reasons such as patient refusal, resection not done, no cancer at surgery, and surgical complications; while six patients randomized to arm B were treated with perioperative 5-FU.

Of the 855 randomized patients, 17 had no pathology reports (12: no cancer at surgery; 3: no resection; and 2: no data). For the 838 patients with pathologic data, 252 were Dukes’ A, B1, or D stage, which removed them from the study. The remaining patients were 289 with Dukes’ B2 and 314 with Dukes’ B3 or C stage disease.

A total of 240 patients with Dukes’ B2 cancer were registered to step 2 (123 arm A, 117 arm B). Two hundred thirty-eight patients with Dukes’ B3, C stage registered to step 2 (114 arm A, 124 arm B) and were to receive adjuvant leucovorin/levamisole and 5-FU.

Baseline demographics and tumor characteristics of the patients with Dukes’ B3, C and Dukes’ B2 stage colon cancer are listed by treatment arm in Table 1. The treatment arms were well balanced in terms of known prognostic factors. The median age was 66 years, about two-thirds of patients were male, and more than 70% of patients were white race. Two-thirds of patients had primary neoplasm at sigmoid colon, cecum, and right colon. More than 90% of patients had documented adenocarcinoma at on-study.

**Table 1:** Baseline demographic and disease characteristics in ITT patients

Variable	B3 and C (*n* = 314)				B2 (*n* = 289)			
	Arm A (*n* = 156)		Arm B (*n* = 158)		Arm A (*n* = 150)		Arm B (*n* = 139)	
	*N*	%	*N*	%	*N*	%	*N*	%
Age at randomization (median, range)	63	30–89	66	25–86	66	28–85	67	31–88
*Sex*								
Male	99	63.5	96	61.2	98	65.3	92	66.7
Female	57	36.5	61	38.9	52	34.7	46	33.3
*Race*								
White	116	74.4	115	72.8	123	82.0	106	76.3
Black	26	16.7	29	18.4	18	12.0	21	15.1
Other		9.0	6	8.9	2	6.0		8.6
*Location of primary neoplasm*								
Cecum	26	16.8	36	22.8	19	12.8	28	20.3
Right colon	26	16.8	36	22.8	32	21.6	26	18.8
Hepatic flexure	4	2.6	5	3.2	16	10.8	9	6.5
Transverse colon	19	12.3	11	7.0	8	5.4	14	10.1
Splenic flexure	6	3.9	3	1.9	8	5.4	10	7.3
Left colon	11	7.1	7	4.4	14	9.5	9	6.5
Sigmoid colon	49	31.6	45	28.5	38	25.7	31	22.5
Rectosigmoid	14	9.0	14	8.9	13	8.8	9	6.5
Multiple primary tumors	0	0.0	1	0.6	0	0.0	2	1.5
Adenocarcinoma histologic type	143	91.7	143	91.1	140	94.0	130	93.5
*Differentiation*								
Well	16	10.3	12	7.7	19	13.0	13	9.6
Moderate	107	68.6	112	72.3	114	78.1	108	79.4
Poor	33	21.2	31	20.0	13	8.9	15	11.0
*Extension of invasion*								
Submucosa but not muscular wall	1	0.7	5	3.2	0	0.0	2	1.4
Muscular wall but not serosa or pericolonic fat	28	18.2	24	15.5	16	10.7	16	11.5
Into serosa or pericolonic fat	97	63.0	83	53.6	127	84.7	117	84.2
Through serosa involving free peritoneal surface	9	5.8	10	6.5	2	1.3	3	2.2
Through serosa or pericolonic fat with adherence to adjacent structures	9	5.8	20	12.9	4	2.7	1	0.7
Beyond serosa or pericolonic fat with invasion of adjacent structures	10	6.5	13	8.4	1	0.7	0	0.0
Presence of obstruction	19	12.5	19	12.3	17	11.5	11	8.1
Perforation at tumor	6	3.9	10	6.4	6	4.1	2	1.5
Number of nodes examined (mean, sd)	14.77	8.3	16.11	10.6	14.19	10.0	14.57	10.3
Number of nodes positive (mean, sd)	3.04	3.8	3	3.4	0	0.0	0	0.0

### Safety

There were no significant differences in postoperative complications (wound infection, abscess, prolonged ileus) within 30 days of surgery, between the patients who received perioperative 5-FU and those randomized to surgery alone without perioperative chemotherapy (Table 2). There were six cases of anastomotic leak or fistulas: three with reoperation and three treated nonoperatively. Four of these patients had no perioperative chemotherapy and two had perioperative chemotherapy. Infusional 5-FU chemotherapy-related adverse events in 418 patients (arm A) were primarily grade 1–2. Using the method of worst degree of toxicity, 47 experienced a maximum grade 3 toxicity and 25 grade 4 toxicity. Anemia, diarrhea, and stomatitis were most frequently reported. As would be expected, there were few toxicities among the 422 arm B patients: three grade 3 and no grade 4 (Table 3).

**Table 2:** Surgical complications

Complication	No perioperative chemotherapy		Perioperative chemotherapy	
	*N*	%	*N*	%
*Wound infection-oper comp*				
Unknown	13	3.04%	15	3.52%
No	395	92.51%	397	92.97%
Yes	19	4.45%	15	3.51%
*Abscess-complictn prim sr*				
Unknown	13	3.04%	14	3.28%
No	409	95.78%	411	96.25%
Yes	5	1.17%	2	0.47%
*Prolonged ileus-complc sr*				
Unknown	13	3.04%	15	3.51%
No	386	90.40%	390	91.33%
Yes	28	6.56%	22	5.15%

**Table 3:** Efficacy outcomes in ITT patients

Efficacy endpoint	Patient population		Arm A	Arm B
Overall survival (OS)	Dukes’ B3/C	Total no. of events/patients	97/156	102/158
		Median OS (years)	10.3 (8.4, 13.2)	9.3 (5.7, 12.3)
		5-Year rate (95% CI)	66.6% (58.5, 73.5)	61.2% (53.0, 68.3)
		10-Year rate (95% CI)	53.6% (45.3, 61.2)	49.1% (40.9, 56.9)
		HR (95% CI) for arm A/arm B	0.88 (0.66, 1.16)	1
	Dukes’ B2	Total no. of events/patients	69/150	71/139
		Median OS	16.1 (13.2, -)	12.9 (10.7, -)
		5-Year rate (95% CI)	85.1% (78.3, 89.9)	78.0% (70.1, 84.1)
		10-Year rate (95% CI)	69.3% (61.0, 76.2)	63.7% (54.8, 71.3)
		HR (95% CI) for arm A/arm B	0.82 (0.59, 1.14)	1
Disease-free survival (DFS)	Dukes’ B3/C	Total no. of events/ patients	55/128	54/131
		Median DFS (years)	6.2 (3.8,-)	7.7 (4.4,-)
		5-Year rate (95% CI)	58.2% (48.4, 66.8)	54.3% (44.2, 63.4)
		HR (95% CI) for arm A/arm B	1.04 (0.71, 1.51)	1
	Dukes’ B2	Total no. of events/patients	38/133	35/123
		5-year rate (95% CI)	74.9% (66.0, 81.8)	72.4% (62.6, 80.1)
		HR (95% CI) for arm A/arm B	0.96 (0.60, 1.53)	1

There were seven deaths during step 1, four attributed to treatment. Of the patients randomized to perioperative 5-FU, two died from sepsis at 2 and 7 days after completing chemotherapy, and a third patient suffered a fatal cardiac arrest on day 3 of infusional 5-FU. All were considered treatment related. Two other patients died from unrelated causes, one on the last day of chemotherapy, and the other 3 weeks after completing chemotherapy. Two deaths occurred in patients randomized to arm B, one from a pulmonary embolus in the immediate postoperative period classified as treatment related. The second patient incorrectly received perioperative 5-FU and died on the last day of the infusion from a treatment-unrelated cause.

After the surgery and perioperative period, the 240 patients with B2 colon cancer received no further adjuvant therapy. Of the 233 patients with Dukes’ B3 and C disease who went on to standard adjuvant therapy, 170 received adjuvant 5-FU/levamisole and 62% completed 12 months of therapy. Sixty-three patients initiated 5-FU/leucovorin and 76% completed six cycles of therapy. Toxicity and patient withdrawal or refusal were the two major reasons for discontinuation of adjuvant chemotherapy. There were four treatment-related deaths, two in patients receiving 5-FU/leucovorin and two receiving 5-FU/levamisole.

### Survival

**Fig. 3:**
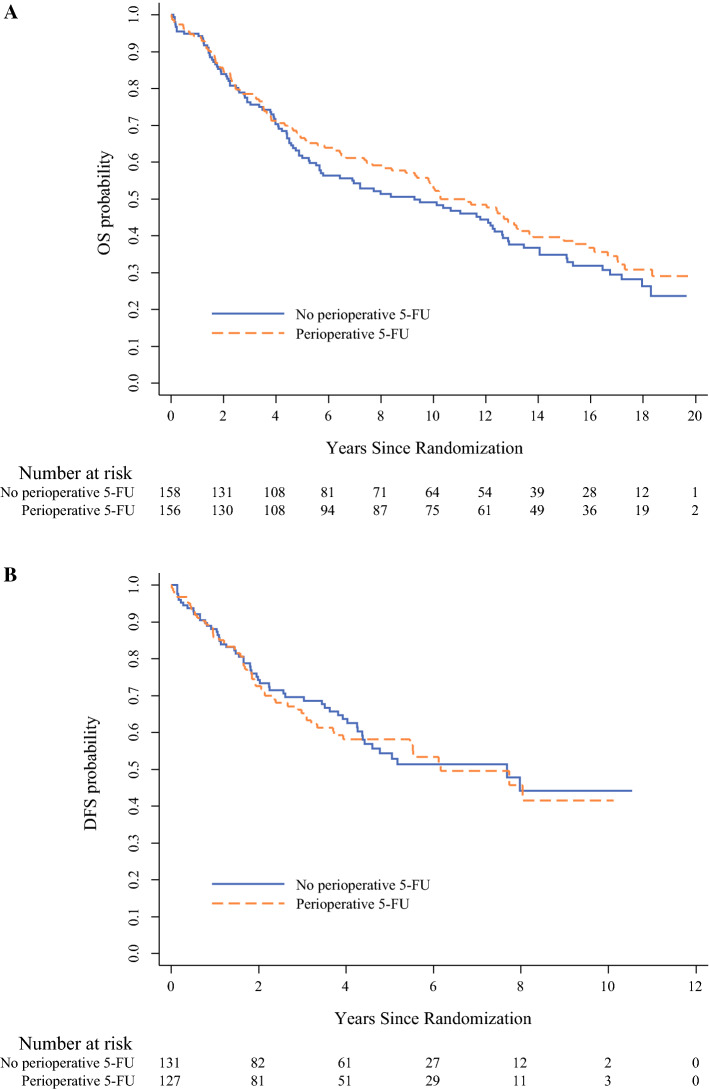
**A** Kaplan–Meier estimates of overall survival for patients with Dukes’ B3 and C disease. There was no significant difference in OS between the two treatment arms, one-sided log rank *p* = 0.178, HR = 0.88 (95% CI 0.66, 1.16). The median OS rate was 10.3 (95% CI 8.4, 13.2) years in patients receiving perioperative 5-FU and 9.3 (95% CI 57.0, 12.3) years in patients without perioperative 5-FU. **B** Kaplan–Meier estimates of disease-free survival for patients with Dukes’ B3 and C disease. There was no significant difference in DFS between the two treatment arms, two-sided log rank *p* = 0.847, HR = 1.04 (95% CI 0.71, 1.51). The 5-year DFS rate was 58.2% (95% CI 48.6, 66.8) in patients receiving perioperative 5-FU and 54.3% (95% CI 44.2, 63.4) in patients without perioperative 5-FU

**Fig. 4:**
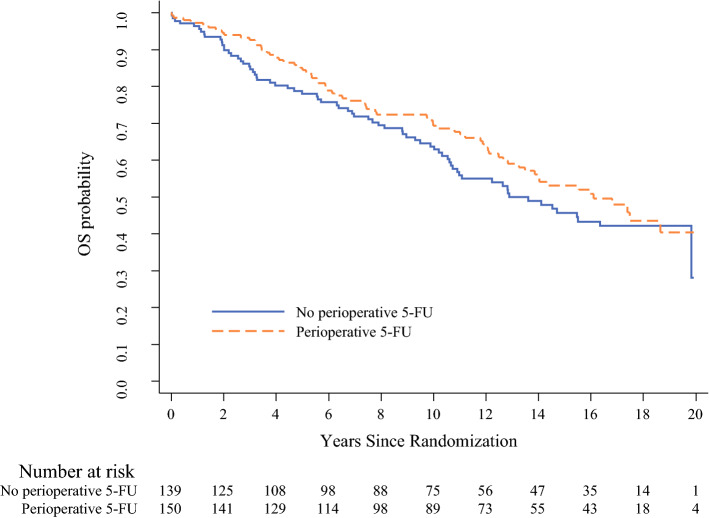
Kaplan–Meier estimates of overall survival for patients with Dukes’ B2 disease. There was no significant difference in overall survival between the two treatment arms, log rank *p* = 0.243, HR 0.82 (95% CI 0.59–1.14). The median OS rate was 16.1 (95% CI 13.2, –) years in patients receiving perioperative 5-FU and 12.9 (95% CI 10.7, –) years in patients without perioperative 5-FU

Efficacy outcomes for OS and DFS comparisons are detailed in Table 3. For patients with Dukes’ B3 and C disease, there was no significant difference in OS between the two treatment arms (one-sided log-rank *p* = 0.178, HR = 0.88, 95% CI 0.66, 1.16, Fig. 3). The median OS was 10.3 years (95% CI: 8.4, 13.2 years) for patients in arm A and 9.3 years (95% CI 5.7, 12.3 years) for patients in arm B (Fig. 3). There was also no significant difference in DFS between the two arms (two-sided log rank *p* = 0.847, HR = 1.04, 95% CI 0.71, 1.51), as shown in Fig. 4. Similarly, for patients with Dukes’ B2 disease, there was no significant difference in OS and DFS between those who did and did not receive perioperative 5-FU (log-rank *p* = 0.243 for OS and *p* = 0.866 for DFS). The median OS was 16.1 years (95% CI 13.2, –) for patients in arm A and 12.9 years (95% CI: 10.7, –) for patients in arm B (Fig. 4).

## Discussion

The concept of perioperative chemotherapy is driven by the hypothesis that the timing of the start of systemic treatment for cancer after surgery is an important determinant of the therapeutic outcome.^1, 8, 9^ The first phase of wound healing (hemostasis and inflammation) occurs immediately after surgery and ends approximately 72 h later. Inflammatory cells migrate to the area of injury, and cytokines and growth factors are released. This triggers the second phase of wound healing (proliferation), which lasts for about 10 days, resulting in the continued migration of cells and the development of a collagen-rich matrix. The third and final phase (remodeling) is when the matrix matures and strengthens further from about day 12 onward. Therefore, the critical aspects of wound healing all occur in the first 2 weeks after tissue injury.

There is evidence that wound healing parallels the ability of cancer cells to metastasize. It has been shown that tumor-derived growth factors orchestrate the development of “premetastatic niches,” which are similar to the matrix of wound healing.^10^ Bone marrow-derived progenitor cells that migrate to sites of tissue injury were also shown to migrate to areas of the “premetastatic niche.”^11^ A reduction in angiogenesis inhibitors and an increased production of oncogenic growth factors, such as transforming growth factor α, follow removal of the primary tumor along with shedding of tumor cells into the circulation. Thus, the wound healing cascade triggered by surgery as studied in animal models appears to assist cancer cells in metastasizing.^12^ Allowing all phases of wound healing to occur prior to the initiation of chemotherapy, such as waiting for 30 days after surgery, may in fact be aiding the cancer cells’ ability to seed metastasis and ultimately compromise patient survival.

This open-label randomized phase III study aimed to investigate the use of perioperative chemotherapy started within 24 h of the completion of surgery for patients with resected Dukes’ B2, B3, and C colon cancer. The study terminated early because of slow accrual and instead of the expected 2000 patients, only 859 enrolled. This study required a coordinated, multidisciplinary team approach so that patients could be consented prior to surgery and perioperative therapy could start within 24 h of resection. Most institutions did not have such a mechanism in place, and many surgeons were not in environments in which this effort could be accommodated; with the passage of time additional regulatory strictures became limiting. Furthermore, there were at least two competing multi-institutional adjuvant trials that opened during the course of this trial which were investigating postoperative adjuvant therapy, and were easier to enroll to because they required less cooperation between treatment modalities.

This trial had limited power to answer the question of the usefulness of perioperative 5-FU. In total, 314 patients with Dukes’ B3 and C disease were enrolled, and this sample size could detect a hazard ratio of 0.625 with adequate power assuming exponential distribution of OS. The trial was revised to have adequate power to detect a 60% improvement in median OS, so it only could detect a big treatment effect. The results did not show significant improvement in OS or DFS in patients receiving perioperative 5-FU therapy compared with patients without such chemotherapy and, thus, the primary endpoint was not met. The study did not report on the location of recurrent disease, nor what was the treatment of the recurrence. In patients with Dukes’ B2 disease, perioperative 5-FU was not associated with statistically significant improvement in either OS or DFS. Yet the overall survival results in the perioperative arms were consistently better for all stages over the 15-year follow-up period.

The study did establish that infusional 5-FU chemotherapy does not impair wound healing in the immediate postoperative period and is generally tolerable. Eighteen percent of patients developed grade 3 or higher treatment-related adverse events, although there were three deaths described as 5-FU treatment related.

Clinically significant results of studies incorporating oxaliplatin established FOLFOX as the current adjuvant chemotherapy standard of care for high-risk colon cancer.^13, 14^ After follow-up of 10 years, the survival rate of patients randomized to oxaliplatin, 5-FU, and leucovoin was 71.7% versus 67.1% for those receiving 5-FU plus leucovorin.^13^

Similarly, the NSABP study that changed practice from adjuvant therapy with 5-FU and levamisole to 5-FU and leucovorin showed a survival difference of 4% in favor of 5-FU/leucovorin (74% versus 70%).^15^ While these chemotherapy trials have led to incremental improvements, the question of the optimal timing of adjuvant chemotherapy remains unanswered. Metaanalyses and retrospective institutional case series show a clear association between the timing of adjuvant chemotherapy and colon cancer survival.^16–19^ A large database study from the UK concluded that the initiation of adjuvant therapy improved survival if it was within 8 weeks of surgery.^20^ Depending on the design of the analysis, worse outcomes are associated with delays in the initiation of adjuvant chemotherapy beyond 4 or 8 weeks after surgery.

No other prospective randomized trial has tested perioperative chemotherapy for colon cancer, yet it remains a highly relevant question today given our advanced understanding of the metastatic process and availability of better systemic therapies, including immune modulators. The conduct of adequately powered, prospective randomized trials is the only way to determine the optimal timing and effects on survival of a perioperative approach to adjuvant therapy.

## Conclusions

The optimal timing of adjuvant chemotherapy following curative colon resection is unknown. Preclinical data and kinetic modeling suggest that surgery can potentiate metastasis and support the notion of initiation of systemic treatment as early as possible. This randomized trial compared perioperative infusional 5-FU for 7 days starting within 24 h of resection with a control group that did not receive perioperative 5-FU. Perioperative 5-FU was well tolerated and did not adversely affect wound healing. This multi-institutional trial was terminated early for slow accrual and found no statistically significant difference in OS or DFS among patients with Dukes’ B2, B3, and C colon cancer.

## Supplementary Information

Below is the link to the electronic supplementary material.

Supplementary file1 (DOCX 17 kb)

Supplementary file2 (PDF 176 kb)
