# Supplementary material for: Phase III Prospectively Randomized Trial of Perioperative 5-FU After Curative Resection for Colon Cancer: An Intergroup Trial of the ECOG-ACRIN Cancer Research Group (E1292)
Source: Ann Surg Oncol. 2022 Oct 28;30(2):1099–109. doi: 10.1245/s10434-022-12705-8 (PMC9807536; doi:10.1245/s10434-022-12705-8)
Supplement: Supplementary file 1 — Supplementary file1 (DOCX 17 kb) [file 10434_2022_12705_MOESM1_ESM.docx]

**Supplement Table 1: Treatment-Related Grade 3-5 Adverse Events on Step 1**

| Toxicity Type | Treatment Arm | | | | | | | |
| --- | --- | --- | --- | --- | --- | --- | --- | --- |
|  | A (n=418) | | | | B (n=422) | | | |
|  | Grade | | | | Grade | | | |
|  |  | 3 | 4 | 5 |  | 3 | 4 | 5 |
|  |  | (n) | (n) | (n) |  | (n) | (n) | (n) |
| Leukopenia |  | 2 | 1 | - |  | 1 | - | - |
| Granulocytopenia |  | 1 | - | - |  | - | - | - |
| Anemia |  | 19 | 3 | - |  | - | - | - |
| Infection |  | 4 | 3 | 2 |  | - | - | - |
| Nausea/vomiting |  | 5 | 2 | - |  | - | - | - |
| Vomiting |  | 2 | 4 | - |  | - | - | - |
| Diarrhea |  | 7 | 7 | - |  | - | - | - |
| Stomatitis |  | 10 | 1 | - |  | - | - | - |
| Liver |  | 1 | 1 | - |  | - | - | - |
| Pulmonary |  |  | 3 | - |  | - | - | - |
| Cardiac |  | 6 | 2 | 1 |  | - | - | - |
| Hypertension |  | 1 | - | - |  | - | - | - |
| Skin |  | 1 | 1 | - |  | - | - | - |
| Neuro-sensory |  | - | - | - |  | 1 | - | - |
| Neuro-motor |  | 1 | 5 | - |  | - | - | - |
| Metabolic |  | 1 | 1 | - |  | - | - | - |
| Others |  | 9 | 5 | - |  | 1 | - | 1 |
| **WORST DEGREE** |  | **47** | **25** | **3** |  | **3** | **-** | **1** |

Note: Adverse events were assessed and graded using the CTCAE V2. Of the 855 randomized patients, 840 patients (including 422 patients on arm B) reported adverse events data and were included in the above table.
