# Supplementary material for: Phase III Prospectively Randomized Trial of Perioperative 5-FU After Curative Resection for Colon Cancer: An Intergroup Trial of the ECOG-ACRIN Cancer Research Group (E1292)
Source: Ann Surg Oncol. 2022 Oct 28;30(2):1099–109. doi: 10.1245/s10434-022-12705-8 (PMC9807536; doi:10.1245/s10434-022-12705-8)
Supplement: Supplementary file 2 — Supplementary file2 (PDF 176 kb) [file 10434_2022_12705_MOESM2_ESM.pdf]

E1292
SWOG 9250
CALGB 9395
NSABP CI63
ACOSOG E1292
EPP E1292
INT 0136

Intergroup Study *Coordinated by the*

EASTERN COOPERATIVE ONCOLOGY GROUP

Phase III Intergroup Prospectively Randomized Trial of Perioperative
5-FU After Curative Resection, Followed by 5-FU/Leucovorin for Patients with Colon Cancer

ECOG STUDY CHAIR
and CALGB STUDY COORDINATOR: M. Margaret Kemeny, M.D.

Rev. 11/93 SWOG STUDY COORDINATOR: Robert P. Whitehead, M.D.

Rev. 3/95,8/99 NSABP STUDY COORDINATOR: Roy Smith, M.D.

Rev. 8/99 ACOSOG STUDY COORDINATOR: Ronald Bleday, M.D.

ECOG STUDY CO-CHAIR: Stanley R. Hamilton, M.D.

Rev. 3/95,8/99 STATISTICIAN: Ronghui Xu, Ph.D.

Rev. 7/96 ECOG PATHOLOGY COMMITTEE CHAIR: Stanley R. Hamilton, M.D.

CALGB DISEASE-ORIENTED
COMMITTEE CHAIR: Robert J. Mayer, M.D.

SWOG DISEASE-ORIENTED
COMMITTEE CHAIR: John S. MacDonald, M.D.

Rev. 3/95 NSABP DISEASE-ORIENTED
COMMITTEE CHAIR: Nicholas J. Petrelli, M.D.

ECOG DISEASE-ORIENTED
COMMITTEE CHAIR: Al B. Benson, III, M.D.

Version Date: March 31, 2010
Update Date: March 31, 2010

<u>STUDY PARTICIPANTS</u>

ECOG Entire Group
Australian GI Trials Group (ECOG)
SWOG Entire Group
CALGB Entire Group
NSABP Entire Group
Rev. 8/99 ACOSOG Entire Group
Rev. 8/99 Expanded Participation Project (Emmes Corporation)

<u>STUDY ACTIVATED</u>

August, 1993

Addendum #1, 11/93: Changed SWOG SC (Cover, Index); Removed ref to tent act date of lab prot E3293 (Schema, Sec. 3.0, Sec. 4.0, Sec. 10.0).

Addendum #2, 6/94: Gender and Race/Ethnic issues; HHS 596 & ADR formsupdated; NCI ADR FAX # added; baseline CXR time frame clarified; typos corrected: (Secs. 1.0, 4.0, 5.3, 11.0, 13.0).

Addendum #3, 3/95: NSABP added as participant (Title, Index, Secs. 3.0,4.0, 4.5, 4.6, 5.32, 11.2), changed Statistician (Title), changed Robert Whitehead, M.D., address (Index), added elig crit for CEA testing time of $\leq$ 2 weeks before reg (Sec. 3.104, 3.304), updated contraception crit (Secs. 3.113, 3.316), updated reg procedures, updated ECOG address (Sec. 4.0), updated DMO zip code (Sec. 5.32), dose mod clarification (Sec. 5.422), added clarifying footnotes to CEA, Colonoscopy, and Preg tests (Sec. 7.0, table), trademark added to Ergamisol (Levamisole, other names) (Sec. 8.2), updated Levamisole incompatibilities (Sec. 8.26), updated Levamisole neurological tox (Sec. 8.28 and App. I), updated path sub (Sec. 10.0, App. III), updated PCO address (Sec. 10.41), changed tense from first person to second, updated Levamisole side effects (App. I), updated CTC table (App. II), updated PCO address and tel number (App. III).

Addendum #4, 9/95: SWOG phone # added (Index page), Corrected typos (Sec. 1.1, 4.2, 9.0, Schema), Clarified numbering (Sec. 3.0), Updated ECC Name + Address (Secs. 4.0, 5.0, 10.4, 10.42, 11.0, App III), clarified Dose Mods (Sec. 5.421).

Addendum #5, 7/96 - CALGB added to ancillary lab study E3293 (Secs. 3.0, 4.0, 7.2, 10.0, 10.2, 10.22, 10.23, 10.41, 10.42, App III), ECOG Path Chair updated (Title, App III), PIN contact updated (Index), checklist added to Sec. 11.0.

Addendum #6, 9/97 - Changed adj chemo from 12 months of 5-FU/Levamisole to 6 months of 5-FU/ Leucovorin; Frozen tissue samples no longer being collected; ECOG PCO address/phone/fax updated; Updated Sec. 5.33; ECOG Fax # added to ADR Sec.; Added and renumbered references; Enrollment in E1295 mentioned as possibility for patients with diarrheal tox; Reregistration directions in schema corrected; Study parameters for Dukes' B3 and C patients moved to below table; Timepoints for flow sheets sub clarified; NSABP SC name and address updated; INT-0089 study number and accrual added; Preop and Postop Eligibility Criteria consolidated; Schema perioperative chemo directions clarified; Objective pertaining to effect of perioperative continuous 5-FU on survival of Dukes' B2 patients elucidated; Statement that Dukes' B2 patients are followed on observation until relapse/death deleted; Reference made to ADR table; References renumbered; Secs. renumbered; Typo corrected; Dukes' stage specified under follow-up time points in Sec 7.0. **REVISED PROTOCOL.**

Addendum #7, 4/98 - Removed thrombocytopenia from listing of potential side effects of Leucovorin (Section 8.210); Typo corrected (Section 9.0); Reformatted Informed Consent to reflect current template (Appendix I); Moved info about tissue testing to new template (Appendix I).

Addendum #8, 8/99 - Schema reformatted (Schema); Added ACOSOG (Cover, Index, Secs. 3.0, 4.0, 4.6, 4.7, 5.35, 5.36, 10.0,11.3); Added EPP (Cover, Index, Secs. 3.0, 4.0, 10.0, App. VI); NSABP Study Coordinator changed (Cover, Index); ECOG Statistician changed (Cover); Address for ECOG and CALGB Study Chair and SWOG Study Coordinator changed (Index); PIN Contact Person changed (Index); AGC $\geq$ 1500/mm^3 changed to WBC $\geq$ 3000/mm^3 (Secs. 3.14, 3.28); CEA levels changed from required to optional (Secs. 3.1142, 3.1152, 7.1 footnote 2); ECOG zip code changed (Secs. 4.0, 5.32, 11.0); Changes to NSABP Registration/Randomization instructions (Sec. 4.5); Changed Grades 1-5 to Grades 4-5 (Sec. 5.32 table); AGC changed to WBC (Secs. 5.41, 7.1, 7.2); "Granulocytopenia (< 1500/mm^3)" changed to "Leukocytopenia (< 3000/mm^3)" (Sec. 5.41); Deleted "SWOG" (Sec. 5.52);Changed "below" to "above" (Sec. 7.2); Deleted reference to red blood cells (App. I, Risks and Discomforts); Deleted "SWOG" and "NSABP" (App. III Memorandum).

Addendum #9, 12/08

Update #1, 3/10

E1292
SWOG 9250
CALGB 9395
NSABP CI63
ACOSOG E1292
EPP E1292
INT 0136
REVISED
Revised 8/99, Addendum #8

INDEX

	Schema
1.0	Introduction
2.0	Objectives
3.0	Selection of Patients
4.0	Randomization/Registration Procedures
5.0	Treatment Plan
6.0	Measurement of Effect
7.0	Study Parameters
8.0	Drug Formulation and Procurement
9.0	Statistical Considerations
10.0	Pathology Review
11.0	Records To Be Kept
12.0	Patient Consent and Peer Judgment
13.0	References
Appendix I	Suggested Patient Consent Form
Appendix II	Common Toxicity Criteria
Appendix III	Pathology Submission Guidelines
Appendix IV	Modified Dukes' Classification
Appendix V	Technique of Colon Resection
Rev. 8/99 Appendix VI	Expanded Participation Project (EPP) Instructions

Rev. 11/93
Rev. 8/99

ECOG AND CALGB STUDY CHAIR
M. Margaret Kemeny, M.D.
University Hospital
Medical Oncology Division
Health Sciences Center, T-17, 080
SUNY at Stony Brook
Stony Brook, NY 11794-8174
Phone: (516) 444-1793
FAX: (516) 444-6348
email: kemeny@surg.som.sunysb.edu

Rev. 3/95,8/99

SWOG STUDY COORDINATOR
Robert P. Whitehead, M.D.
University of Texas Medical Branch
Division of Hematology/Oncology
301 University Boulevard
Galveston, TX 77555-0565
Phone: (409) 772-1164
FAX: (409) 772-3533
email: rpwhiteh@utmb.edu

Rev. 3/95, 7/96, 9/97, 8/99

NSABP STUDY COORDINATOR
Roy Smith, M.D.
East Commons Professional Building
Four Allegheny Center - 5th Floor
Pittsburgh, PA 15212-5234
Phone: (412) 330-4600
FAX: (412) 330-4660

Rev. 8/99

ACOSOG STUDY COORDINATOR
Ronald Bleday, M.D.
Colorectal Surgery
Beth Israel Deaconess Medical Center
110 Francis Street, Suite 3A
Boston, MA 02215
Phone: (617) 632-0947
FAX: (617) 632-7424
email: rbleday@caregroup.harvard.edu

Rev. 8/99

PROTOCOL INFORMATION NETWORK CONTACT PERSON
Pat Hentschel, N.P.
University Hospital
Medical Oncology Division
Health Sciences Center, T-17, 080
SUNY at Stony Brook
Stony Brook, NY 11794-8174
Phone: (516) 444-7863
FAX: (516) 444-6389
email: phentsch@mail.som.sunysb.edu

Rev. 8/99

EXPANDED PARTICIPATION PROJECT CONTACT PERSON
Gity Nasim
The Emmes Corporation
11325 Seven Locks Road - Suite 214
Potomac, MD 20854
Phone: (301) 299-8655
email: gnasim@emmes.com

Rev. 8/99

SCHEMA

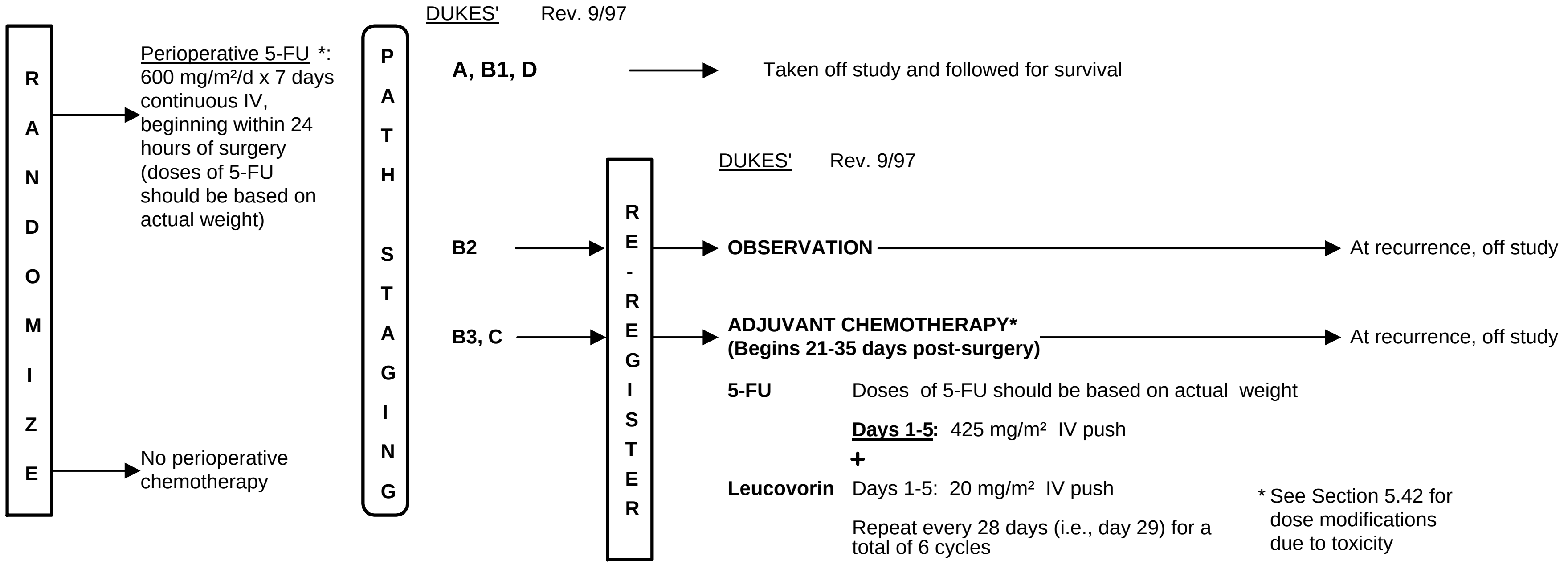

RANDOMIZATION:

Patients will be randomized pre-operatively within 2 weeks of surgery, or post-operatively within 24 hours of surgery, for primary colon cancer with no known metastases. If metastatic disease is found during surgery, or if there is unresectable residual disease after surgery, the patient will be taken off-study, if already randomized, and followed for survival.

PATHOLOGIC STAGING: (within 2 weeks after surgery)

All patients staged with Dukes' A, B1, and D disease will be taken off study and followed for survival. Those patients staged with Dukes' A, B1, and D disease who were randomized to receive perioperative 5-FU will have this perioperative 5-FU discontinued. Patients staged with Dukes' B2, B3, or C disease must be re-registered (see below).

Rev. 9/97

RE-REGISTRATION: (Within 35 days after surgery)

Dukes' B2 patients must be re-registered to observation. Dukes' B3 and C patients must be re-registered to adjuvant chemotherapy with 5-FU plus leucovorin. Dukes' A, B1 and D patients will not be re-registered.

SUBMISSION OF PATHOLOGY MATERIALS: SLIDES/PARAFFIN BLOCKS = within 1 month after surgery)

Rev. 5/96, 9/97

The submission of paraffin blocks and H&E slides of tumor and normal colonic mucosa is required for ECOG patients entered on this study. See Section 7.2 and 10.0 for details.

Rev. 11/94, 5/96

**NOTE:**	**When randomizing ECOG and CALGB patients to E1292/C9395, it is strongly encouraged that they be simultaneously registered to laboratory companion protocol E3293**

1.0 INTRODUCTION

Over 110,000 new cases of colon carcinoma are diagnosed every year in the United States. Over 50,000 of these patients will die of this disease. Thus, approximately 45% of patients who get colon carcinoma will die of distant metastases or, more rarely, local recurrence (1). The purpose of this study is to look at the effects of intravenous perioperative 5-FU therapy, followed by adjuvant
Rev. 9/97 5-FU/leucovorin, on the disease-free interval and survival for colon cancer. Studies that support this therapy are cited below, and involve either intravenous or portal vein perioperative 5-FU therapy.

1.1 Adjuvant Chemotherapy

Because of the high incidence of hepatic metastases in patients who develop metastatic disease, the NSABP carried out a perioperative adjuvant protocol using a 7 day infusion of 5-FU 600 $mg/m^2/d$ into the portal vein starting the day of surgery (2). This protocol was conducted in response to the data from Ian Taylor which suggested that portal vein infusion significantly decreased the incidence of liver metastases (3). The NSABP C-02 study was conducted from March 1984 to July 1988 with accrual of 1158 patients. This study required a pre-operative randomization, thus patients who were found to have metastases at surgery (Dukes' D) were not eligible. Of the 1158 patients, 152 patients were found to be Dukes' D at operation. In this study all other patients were treated, whether Dukes' A, B or C, because pathology could not be obtained before the portal infusion was well under way. For the control group of patients, 24.9% turned out to have a Dukes' A classification while 21.9% of the 5-FU infusion group had Dukes' A. The continuous infusion of 600 $mg/m^2/d$ of 5-FU was very well tolerated with no significant increases in wound infections, wound dehiscence or anastomotic leaks. Of the 577 patients randomized to the portal infusion, 44 had the infusion discontinued, but in only 20 of these was the cessation of therapy secondary to toxic side effects. The preliminary results of this study have shown no advantage as far as the development of liver metastases for the group receiving the portal infusion. However, when disease free survival was studied, there was a significant benefit at 5 years for patients in the infusion arm ($p = .01$). The survival curves also reflected this benefit for 5 year survival ($p = .01$) (4).

Another randomized trial from England has recently published a similar result. In this study 130 patients were randomized to intraportal 5-FU plus heparin started during surgery, another 123 patients received intraportal heparin and another 145 patients received no postoperative treatment. There was no difference in the incidence of liver metastases within the groups but patients who had been treated with 5-FU had a significant survival advantage ($p< .03$).

This study and the NSABP study came to the same conclusion: the regional approach of adjuvant chemotherapy through the portal vein does not lower the incidence of liver metastases but the perioperative portal infusion of 5-FU does increase survival in Dukes' C patients (5). Both the NSABP study and the English study found that the perioperative chemotherapy seemed to have an effect after 3 years of follow-up. The majority of adjuvant trials in which chemotherapy was given post-operatively at least a week later show more immediate outcome improvement. This temporal difference in outcome improvement may be explained by postulating different mechanisms of action of these 2 adjuvant approaches. If that is correct, a combination of the two approaches may be beneficial.

There are several trials in progress assessing the efficacy of different regimens of intravenous adjuvant chemotherapy, all including 5-FU and some modulator. In October1989, the North Central Cancer Treatment Group (NCCTG) and the Mayo Clinic published a report indicating a disease-free survival benefit for levamisole plus 5-FU ($p = .02$) when given as adjuvant therapy
Rev. 9/95 for colon cancer. This difference was observed predominantly in the Dukes' C patients. There was also a survival benefit but only in the Dukes' C patients (6).

An intergroup study was then performed and reported in 1990 (7) and updated in 1992 (8). This study randomized over 1200 patients with Dukes' B2 or C colon cancer to receive post-operative adjuvant therapy with levamisole alone, levamisole plus 5-FU or observation. The follow-up time was 5 years. There were 168 recurrences in the observation arm, 162 with levamisole alone, and 112 with levamisole + 5-FU. Deaths were 153, 144, and 108 respectively. Levamisole plus 5-FU decreased cancer related deaths by 32% ($p < 0.004$). The toxicity was low for this regimen; therefore, the authors recommended that 5-FU plus levamisole should be the standard for adjuvant chemotherapy in patients with Dukes' C colon cancer.

The mechanism of action for levamisole is not clearly understood. Levamisole alone did not have an effect on survival in the adjuvant setting. No single immunomodulatory effect of levamisole can be specified that would account for its beneficial results when combined with 5-FU. Levamisole has been studied extensively "in vitro." It has been shown to increase chemotaxis of monocytes and to restore defective neutrophils among other activities. Levamisole seems to be more effective "in vivo" than can be proved by the "in vitro" studies. This may be because levamisole is acting indirectly, such as increasing the production of a lymphokine which would be tumorocidal. Also, levamisole may restore immune functions in patients with cancer or post surgery that cannot be detected in the laboratory situation. The exact mechanisms of action have yet to be elucidated (9).

In 1982, a randomized study was begun in Australia and New Zealand. Patients with colon cancer were randomized pre-operatively into one of 3 treatment arms:

1. 5-FU 600 mg/m^2/d for 7 days immediately following surgery given via a catheter into the portal circulation.

2. 5-FU 600 mg/m^2/d for 7 days immediately following surgery given into the systemic venous circulation.

3. No post-operative chemotherapy.

A total of 372 patients were randomized as of 1987. Of these, 232 were analyzable and 140 were ineligible (25 no cancer, 40 Dukes' A, 75 Dukes' D, 35 no follow-up).

The post-operative chemotherapy was well tolerated. The study at this time suggests that patients with portal vein chemotherapy have improved survival, but follow-up is not long enough to make any conclusions since the study was ongoing at the time of the report. However, this study has provided evidence that immediate post-operative 5-FU can be given
Rev. 9/97 safely with a schedule of 600 mg/m^2/d for 7 days (10,11).

Rev. 9/97 In 1992, the large intergroup study of adjuvant chemotherapy for colon cancer, INT-0089, was closed after accruing 3,794 patients. This study compared the standard chemotherapy regimen of 5-FU + Levamisole for 12 months to 3 other treatments:

1. Low dose Leucovorin + 5-FU
2. High dose Leucovorin + 5-FU
3. Low dose Leucovorin + 5-FU + Levamisole

All 3 regimens are given for 6 months.

In 1996, an interim analysis demonstrated no significant differences in the disease-free survival or overall survival for any of the arms. This was reported at the ASCO meeting in
Rev. 9/97 May, 1996 (12). Because there were no differences in outcome and because the duration

Rev. 9/97 of the treatment for all the leucovorin arms was 6 months vs. the 12 months length of the 5-FU/Levamisole arm, the 5-FU/low dose leucovorin arm has emerged as the obvious choice for treating patients in the community. Further close analysis and follow-up of greater than Rev. 9/97 4 years has held up these findings (13).

The study proposed in this concept combines the perioperative approach with the now Rev. 9/97 standard adjuvant chemotherapy for colon cancer, 5-FU and leucovorin. Since all the previous studies mentioned have shown a benefit for Dukes' C patients, all Dukes' C patients will receive the 5-FU/leucovorin. The proper treatment for Dukes' B patients is not as clear since many studies did not show a statistically significant benefit for this group. Because of Rev. 9/97 these data, the Dukes' B2 patients will be randomized for the perioperative part of this study only. They will not receive the post-operative 5-FU/leucovorin.

For the B3 patients, although no study has proven a benefit for adjuvant chemotherapy, it is also true that the studies have not been large enough to look specifically at the subgroup.

Because of the poorer survival of this group it is felt they should be treated in the same fashion as the C patients and be eligible for both stages of the protocol.

1.2 <u>Prognostic Markers</u> (see laboratory companion protocol, E3293, for details)

The chromosomal and molecular genetic alterations in colorectal cancer are beginning to be defined. It seems clear that a number of oncogenes (which are tumor promoting genes) and tumor suppressor genes (which restrain tumor growth) contribute to the development of colorectal cancer (9).

Allelic losses have been observed on almost all chromosomal arms in colorectal cancer with some of the alterations seen more consistently than others. The most common sites for these losses are on chromosomes 1, 5, 8, 17, and 18. Tumor suppressor genes have been localized to some of these chromosomes, such as the APC (Adenomatous Polyposis Coli) gene and MCC (mutated in colorectal cancer) gene on 5q, the p53 gene on 17p and Rev. 6/94 DCC (deleted in colorectal cancer) gene on 18q.

Understanding of the clinical utility of the identified genetic alterations is in its infancy. Initial data from three series of patients suggest that allelic deletions involving the p53 Rev. 6/94, 9/97 gene on chromosome 17p (14) and the DCC gene on chromosome 18q (15) may be of prognostic value. There is, however, conflicting evidence from these studies as to the prognostic value of the deletions. Only 17p allelic deletion was found to be associated with poor prognosis in one study, while only 18q deletion was associated with poor Rev. 6/94, 9/97 survival in another study (14, 15). A large prospective study is needed to answer the question of validity of these markers for general clinical usage. Furthermore, no study has addressed the issue of chemotherapy response in relation to molecular genetic alterations. Laboratory companion protocol, E3293, will provide us with over 1000 tumor specimens to define the role of p53 and DCC in the prognosis of colon cancer.

Furthermore, it is not known if patients who benefit from chemotherapy have a different chromosomal pattern than those who do not respond. This laboratory study will also provide a prospective trial of the chromosomal patterns present in patients and could be compared with the effectiveness of chemotherapy in those patients.

Rev. 6/94 1.3 Gender and Racial/Ethnic Issues in E1292

1.31 Evidence For/Against Treatment by Gender Interactions in the Effects of Perioperative 5-Flourouracil

To our knowledge there is no literature addressing this issue for perioperative treatment. More generally, there has been little attention paid to gender differences with respect to treatment, and no ECOG colon study has shown any gender related interactions with adjuvant therapy. A review of recent papers reporting the effects of postoperative 5-FU in colorectal cancer shows three studies suggesting possible gender interactions with treatment, but they are in conflict with regard to the direction
Rev. 9/97 of effect. A 1988 NSABP rectal study (16) showed increased disease-free survival of MOF over no adjuvant treatment in males but the corresponding 1988 colon
Rev. 9/97 protocol (17), using the same MOF schedule, showed no gender interaction. A 1989
Rev. 9/97 NCCTG colorectal study (18) reported an increase in disease-free survival in females for their 5-FU plus levamisole arm in a subset analysis. In contrast, a second NCCTG study in colon cancer conducted in 1990 (7) showed, in subset analysis, a differential benefit in males due to 5-FU plus levamisole. In addition, a
Rev. 9/97 recent paper on 5-FU clearance in head and neck cancer (19) demonstrated lower clearances in females but the doses examined in that study were higher than those used in this colon study (500 used in this colon study (500 to 1,000 mg/m^2). Other
Rev. 9/97 recent colorectal studies involving 5-FU (20-23) reported no gender by treatment interactions. It is important to interpret the conflicting results of the subset analyses cautiously, since exploratory data investigations can often lead to spurious associations that would not be reproducible.

Thus at present there appears to be no strong evidence for a gender by treatment effect involving 5-FU used as a postoperative adjuvant in colon cancer, and no indication that treatment differences may exist in the perioperative setting.

1.32 Evidence For/Against Treatment by Racial/Ethnic Interactions in the Effects of Perioperative 5-Flourouracil

We are aware of essentially no literature addressing this issue for 5-FU therapy or for any other adjuvant therapy in colorectal cancer, as the issue of different effects within racial/ethnic groups has received little attention in this disease. Thus at present there is no evidence either for or against the possibility of racial/ethnic group by treatment effect interactions for this study.

1.33 Gender and Racial/Ethnic Representation

Based on accrual to the recent intergroup colon adjuvant trial, E2288, we expect roughly 55% of the cases on E1292 to be male and 45% to be female; 86% of cases to be white, 2.1% to be hispanic, 10% to be black (non-hispanic), 1.2% to be Asian/Pacific Islander, and <0.5% to be native American. The proportion of minorities could be somewhat higher, because of general efforts in ECOG to improve minority accrual on clinical trials. These efforts include conducting focus groups/workshops with community physicians who are not currently affiliated with a clinical cooperative group (especially physicians who treat a large number of minority cancer patients), and developing and implementing a marketing and outreach intervention to increase minority accrual.

1.34 Design Considerations/Analysis Plans

In general there are no strong reasons to expect differential effects of 5-FU therapy in gender and minority subgroups, especially in the perioperative colon setting. The fact that studies reporting on gender effects are in conflict as to the direction of any treatment benefit leads to the possible conclusion that subset analyses may not be reproducible and therefore it may not be prudent to modify accrual objectives for this perioperative study based on them. Furthermore, there is no information to date on racial/ethnic differences in response to adjuvant treatment for colon cancer in general. Thus the study will also not have separate accrual targets for these subgroups.

Treatment assignment in the study will not be stratified on gender or racial/ethnic status since the randomization should automatically lead to reasonable balance in treatment assignments within gender and racial/ethnic subgroups. In the final analysis, the effect of therapy will be examined separately with these subgroups by plotting estimated disease-free survival and overall survival curves by treatment separately by gender and racial/ethnic subgroups. Tests for the effect of perioperative therapy within these groups will be conducted using log rank tests and by modeling treatment effects by gender and racial/ethnic group interactions in proportional hazards models.

2.0 OBJECTIVES

Rev. 9/97

2.1 To determine if adjuvant therapy with one week of continuous 5-FU given within 24 hours of a curative colon resection followed by 6 months of 5-FU/leucovorin is effective in prolonging the disease free interval and increasing survival in patients with Dukes' B3 or C colon cancer,when compared to patients who are treated with 5-FU/leucovorin only. Endpoints include: treatment failure--as defined by recurrence of local/regional or distant metastases--and survival.

Rev. 9/97

2.2 To determine if a week of perioperative continuous 5-FU affects disease-free survival and survival in patients with Dukes' B2 colon cancer.

2.3 To establish within ECOG a Central Tissue Repository for paraffin blocks and a frozen tissue bank.

The following objectives apply to companion laboratory protocol, E3293:

2.4 To confirm studies performed in smaller groups of patients to show that 17p deletion and 18q deletion are prognostic markers in a randomized setting.

2.5 To compare the response to chemotherapy in the different subgroups according to their chromosomal deletion profile.

3.0 SELECTION OF PATIENTS

Randomization will occur ≤ 2 weeks before the operation or within 24 hours after the operation, after consent is obtained. ***No exceptions will be allowed.*** Patients found to have residual disease or intraoperative metastatic disease outside of the draining lymph nodes will not receive the randomized treatment and will be treated off-study at the discretion of the participating investigator.

Rev. 9/97

If the patient is randomized PRE-OPERATIVELY, see Sections 3.1 and 3.2 for eligibility criteria. If the patient is randomized POST-OPERATIVELY, see Sections 3.1 and 3.3 for eligibility criteria.

Rev. 11/93, 3/95, 7/96, 8/99

NOTE: The submission of pathology material is required for <u>ECOG patients and strongly encouraged for CALGB patients</u> entered on this protocol (see Sections 7.2 and 10.0). Submission of tissue samples is <u>not</u> required for SWOG, ACOSOG, EPP or NSABP patients.

When randomizing <u>ECOG or CALGB</u> patients to E1292/C9395, it is strongly recommended that they be simultaneously registered to laboratory companion protocol E3293.

Rev. 9/97

Rev. 9/95, 9/97

3.1 <u>Eligibility Criteria</u>

3.11 Must have adenocarcinoma of the colon documented by colonoscopy or barium enema.

3.12 Must not have a dual primary, as revealed by colonoscopy or barium enema.

3.13 Must start perioperative chemotherapy, if assigned, within 24 hours of the end of surgery.

Rev. 8/99

3.14 Have WBC $\geq$ 3000/mm^3 and platelets $\geq$ 100,000/mm^3, $\leq$ 2 weeks prior to randomization.

3.15 Have adequate renal (serum creatinine $\leq$ 2.0 mg/dl) and hepatic function (bilirubin $\leq$ 2.0mg/dl), within two weeks prior to randomization.

3.16 Have ECOG Performance Status of 0, 1 or 2 within 2 weeks prior to randomization.

3.17 No concurrent radiation or chemotherapy, prior exposure to 5-FU, or prior radiation or chemotherapy for this malignant disease.

3.18 No concurrent second malignant disease or any previous malignant tumor within the past 5 years except superficial squamous or basal cell carcinoma of the skin or *in situ* carcinoma of the cervix.

3.19 No history of non-malignant systemic disease that would preclude the chemotherapy treatment option.

3.110 Not pregnant or lactating.

Rev. 3/95

3.111 Women of childbearing potential and sexually active males should use an accepted and effective method of contraception.

3.112 Must be $\geq$ 18 years of age.

3.113 Give written informed consent.

3.114 <u>For Patients Randomized Pre-operatively</u>

3.1141 Must be randomized to the study no more than 2 weeks pre-operatively.

Rev. 3/95, 8/99

Rev. 9/95 3.115 For Patients Randomized Post-operatively

3.1151 Must be randomized to the study within 24 hours post-operatively so that the chemotherapy, if assigned, can begin no later than 24 hours after surgery.

Rev. 3/95, 8/99

Rev. 8/99 3.1152 Complete resection must have been performed with no evidence of residual disease or distant metastasis.

Rev. 8/99 3.1153 Distal margin of the tumor must not extend below the peritoneal reflection in the area of the rectum.

Rev. 8/99 3.1154 Single primary colon carcinoma without free perforation demonstrated. Patients with intestinal obstruction are eligible. Preliminary or complementary colostomy **does not** preclude entry of a patient.

Rev. 9/95 3.2 Eligibility Criteria for Re-Registration (for patients randomized pre-operatively)

All Dukes' B2, B3, and C patients must be re-registered < 35 days post-op, after histopathological confirmation of tumor and Dukes' staging classification. Dukes' A, B1, and D patients will be removed from the study and will be treated at the discretion of their physician. Survival data will continue to be collected on Dukes' A, B1, and D patients until death.

Rev. 9/97 Dukes' B2 patients will have perioperative 5-FU ***continued*** to completion, if so assigned, but will receive NO further adjuvant treatment.

Rev. 9/97 Patients with Dukes' B3 and C disease must begin adjuvant 5-FU/leucovorin treatment between 21 and 35 days following surgery.

3.21 Must have pathologic classification of Dukes' B2, B3, or C disease (see Appendix IV for classification details) by the contributing institution.

3.22 Must be re-registered < 35 days after surgery.

3.23 Have ECOG Performance Status of 0, 1 or 2.

3.24 Complete resection must have been performed with no evidence of residual disease or distant metastasis.

3.25 Distal margin of the tumor must not extend below the peritoneal reflection in the area of the rectum.

3.26 Single primary colon carcinoma without free perforation demonstrated. Patients with intestinal obstruction are eligible. Preliminary or complementary colostomy **does not** preclude entry of a patient.

3.27 Started perioperative 5-FU, if assigned, within 24 hours of surgery.

For Dukes' B3 and C patients only:

Rev. 9/97, 8/99 3.28 Have WBC $\geq$ 3000/mm^3 and platelets $\geq$ 100,000/mm^3, $\leq$ 1 week prior to beginning adjuvant 5-FU/leucovorin therapy.

3.29 Have adequate renal (serum creatinine $\leq$ 2.0) and hepatic function (bilirubin $\leq$ 2.0), within one week prior to beginning chemotherapy.

Rev. 9/95, 9/97 3.3 Eligibility Criteria for Re-Registration (for patients randomized post-operatively)

All Dukes' B2, B3, and C patients must be re-registered $\leq$ 3 5 days post-op, after histopathological confirmation of tumor and Dukes' staging classification. Dukes' A, B1, and D patients will be removed from the study and will be treated at the discretion of their physician. Survival data will continue to be collected on Dukes' A, B1, and D patients until death.

Dukes' B2 patients will have perioperative 5-FU ***continued*** to completion, if so assigned, but will receive NO further adjuvant treatment. At the time of re-registration, Dukes' B2 patients will be followed on observation until relapse or death.

Rev. 9/97 Patients with Dukes' B3 and C disease must begin adjuvant 5-FU/leucovorin treatment between 21 and 35 days following surgery.

3.31 Must have pathologic classification of Dukes' B2, B3, or C disease (see Appendix IV for classification details) by the contributing institution.

3.32 Patient must be re-registered $\leq$ 35 days after surgery.

3.33 Have ECOG Performance Status of 0, 1 or 2.

3.34 Started perioperative 5-FU, if assigned, within 24 hours of surgery.

For Dukes' B3 and C patients only:

Rev. 9/97, 8/99 3.35 Have WBC $\geq$ 3000/mm^3 and platelets $\geq$ 100,000/mm^3, $\leq$ 1 week prior to beginning adjuvant 5-FU/leucovorin therapy.

3.36 Have adequate renal (serum creatinine $\leq$ 2.0) and hepatic function (bilirubin $\leq$ 2.0), within one week prior to beginning chemotherapy.

4.0 RANDOMIZATION/REGISTRATION PROCEDURES

Rev. 6/94, 3/95 **A signed HHS 310 Form, a copy of the institution's IRB-approved informed consent document, and written justification for any changes made to the informed consent for this**
Rev. 9/95 **protocol must be on file at the ECOG Coordinating Center before an ECOG institution may enter patients. The signed HHS 310, institution's informed consent, and investigator's justification for changes will be submitted to the following address:**

Rev. 3/95, 9/95 **ECOG Coordinating Center**
Frontier Science
ATTN: IRB
303 Boylston Street
Rev. 8/99 **Brookline, MA 02445-7648**
FAX (617) 632-2990

Patients must not start protocol treatment prior to registration.

Rev. 3/95, 7/96, 8/99

NOTE: The submission of pathology material is required for ECOG patients and strongly encouraged for CALGB patients entered on this protocol (see Sections 7.2 and 10.0). Submission of tissue samples is not required for SWOG, ACOSOG, EPP or NSABP patients.

Dr. Stanley Hamilton's assistant, Mrs. Rahj Robinson, will be notified at time of perioperative randomization, and will then alert the Pathology Department of the institution in which the patient will undergo/has undergone the operation. Rev. 11/93

Rev. 7/96 **When randomizing ECOG or CALGB patients to E1292 (C9395), it is strongly recommended that they be simultaneously registered to laboratory companion protocol E3293.**

For SWOG patients, the investigator will follow the instructions in Section 4.3.
For CALGB patients, the investigator will follow the instructions in Section 4.4.
Rev. 3/95 **For NSABP patients, the investigator will follow the instructions in Section 4.5.**
Rev. 8/99 **For ACOSOG patients, the investigator will follow the instructions in Section 4.6.**

4.1 ECOG Randomization

Rev. 9/95, 9/97 To randomize a patient, the investigator will telephone the Central Randomization Desk at the ECOG Coordinating Center at (617) 632-2022. For re-registration of Dukes' B2 patients to observation and Dukes' B3 or C patients to adjuvant 5-FU/leucovorin, see Section 4.2)

Rev. 9/95 The following information will be requested by the ECOG Coordinating Center:

4.11 Protocol Number

4.12 Investigator Identification

4.121 Institution name and/or affiliate

4.122 Investigator's name

4.13 Patient Identification

4.131 Patient's name or initials and chart number

4.132 Patient's Social Security number

4.133 Patient Demographics

4.1331 Sex

4.1332 Birthdate (MM/YY)

4.1333 Race

4.1334 9 Digit Zip Code

4.1335 Method of Payment

4.14 Eligibility Verification

Patients must meet all of the eligibility requirements listed in Section 3.1 OR 3.3. The randomization specialist will verify eligibility by asking questions from the STEP 1 eligibility checklist, which has been appended to the protocol. A
Rev. 9/95 confirmation of treatment assignment will be forwarded by the ECOG Coordinating Center.

4.2 ECOG Re-registration after Histopathological Staging

NOTE: The contributing institution's pathology department must classify the colon
Rev. 9/95 **cancer as Dukes' A, B1, B2, B3, C, or D within 2 weeks of the operation. Dukes' A, B1, and D patients will go off study; Dukes' B2, B3, and C patients must be re-registered as indicated below.**

To register a patient, the investigator will telephone the Central Randomization Desk at the
Rev. 9/95 ECOG Coordinating Center at (617) 632-2022. The following information will be requested:

4.21 Protocol Number

4.22 Investigator Identification

4.221 Institution name and/or affiliate

4.222 Investigator's name

4.23 Patient Identification

4.231 Patient's name or initials and chart number

4.232 Patient's Social Security number

4.233 Patient Demographics

4.2331 Sex

4.2332 Birthdate (MM/YY)

4.2333 Race

4.2334 9 Digit Zip Code

4.2335 Method of Payment

4.24 Eligibility Verification

Patients must meet all of the eligibility requirements listed in Section 3.2 OR 3.4. The randomization specialist will verify eligibility by asking questions from the STEP 2 eligibility checklist, which has been appended to the protocol. A confirmation of
Rev. 9/95 registration will be forwarded by the ECOG Coordinating Center.

4.3 SWOG Registration / Randomization

Investigators will call the Southwest Oncology Group Statistical Center at (206) 667-4623 between the hours of 7:00 a.m. and 2:30 p.m. (PT) Monday through Friday excluding holidays. The Statistical Center will obtain and confirm all eligibility criteria and information as per Sections 4.1 and 4.2. In addition the Statistical Center will request the date informed consent was obtained and the date of IRB approval for each entry. The Statistical Center will
Rev. 9/95 then contact the ECOG Coordinating Center to register and randomize the patient after which the Statistical Center will contact the Institution to confirm registration and relay the treatment
Rev. 9/95 assignment for that patient. The ECOG Coordinating Center will forward a confirmation of treatment assignment to the Statistical Center for routing to the participating Institution.

4.4 CALGB Registration/Randomization

CALGB randomization will be accepted through the Main Institution only, prior to initiation of therapy. Confirm all selection criteria listed in Section 3.0. Call the CALGB Data Management Center (919-286-4704, Monday to Friday, 9:00 a.m. to 5:00 p.m. Eastern Time).
Rev. 9/95 The CALGB Registration Desk will then contact the ECOG Coordinating Center to register and randomize the patient, after which the CALGB office will contact the Institution to confirm
Rev. 9/95 registration and relay the treatment assignment for that patient. The ECOG Coordinating Center will forward a confirmation of treatment assignment to the CALGB Office for routing to the participating Institution.

Rev. 3/95,8/99 4.5 NSABP Registration/Randomization

Note: A signed 310 form, indicating current IRB approval for the study, must be on file at the NSABP Biostatistical Center before any NSABP institution may enter a patient.

NSABP Institution Investigators or Data Coordinators must fax a completed Eligibility Checklist and a signed, dated, and witnessed Consent Form to the NSABP Biostatistical Center at (412) 383-2065 between the hours of 8:30 a.m. and 4:00 p.m. (eastern time) Monday through Friday excluding holidays. Senders must include a telephone number/fax number on the cover sheet so they may be contacted after the NSABP receives the randomization information from ECOG. The randomization specialist at the NSABP Biostatistical Center will confirm the eligibility and patient selection criteria and will then contact the ECOG Coordinating Center to register and randomize the patient. The NSABP randomization specialist will then contact the institution to confirm registration and relay the treatment assignment for that patient. The ECOG Coordinating Center will forward a confirmation of treatment assignment to the NSABP Biostatistical Center for routing to the participating institution. The entire randomization procedure must be completed in time for the patient to begin therapy no later than 24 hours after surgery; therefore, it is important that an institution Investigator or Data Coordinator fax the necessary information to the NSABP Biostatistical Center as soon as possible after the patient's surgery, when randomizing post-operatively.

Rev. 8/99 4.6 ACOSOG Registration/Randomization

A signed HHS 310 Form, a copy of the institution's IRB-approved informed consent document, and written justification for any changes made to the informed consent for this protocol must be on file at the ACOSOG Coordinating Center before an ACOSOG institution may enter patients (see SOP:IRB). These will be submitted to:

American College of Surgeons Oncology Group
ATTN: Cherry R. Rallonza
633 N. Saint Clair Street
Chicago, IL 60611-3211
Phone: (312) 202-5312
Fax: (312) 202-5011
E-mail: trallonza@facs.org

The IRB approval date and Form 1572 date must be within one year of patient entry into the study. Patients must not start protocol treatment prior to registration. Treatment should start within three working days of registration.

To register eligible patients on study, the registering site will access the ACOSOG web page at http://www.acosog.org.

The site will be asked for the following information: Study Number registering for (E1292), institution identifier, and investigator identifier. All of the above information are required for the registration process to continue. Once the above information have been verified, further registration instructions will be provided to the registering site to complete the registration process and get the randomization assignment. The registration process can likely be completed in 15 minutes or less under normal circumstances.

Rev. 8/99 4.7 Cancellation Guidelines

If a patient does not begin assigned treatment, the patient may be canceled. Reasons for cancellation should be submitted in writing to the following offices as soon as possible: For
Rev. 9/95 ECOG patients, to the ECOG Coordinating Center (ATTN: DATA); for CALGB patients, to the CALGB Central Office. Data will be collected on all canceled patients (see Section 11.0). **Note:** A patient may only be canceled if no protocol therapy is administered. Once a patient has been given protocol treatment, all forms should be submitted.

For SWOG patients, the SWOG Statistical Center will not consider a patient canceled.

Rev. 3/95 For NSABP patients, the NSABP Biostatistical Center will not consider a patient canceled.

Rev. 8/99 For ACOSOG patients, the ACOSOG Statistical Center will not consider a patient canceled.

5.0 TREATMENT PLAN

5.1 Surgical Guidelines

5.11 Resection

Please refer to Appendix V: *Technique of Colon Resection*. The colonic tumor must be resected in its entirety. The tumor must not extend below the peritoneal reflection in the area of the rectum. The distal and proximal bowel end must be greater than 5 cm from the tumor. The draining lymph nodes must be removed "en bloc" with the colon specimen. The colon specimen should be sent **immediately** to Pathology upon its removal (see Section 10.0).

- Primary anastomosis or colostomy will be at the discretion of the operating surgeon.
- The procedure must be done as a laparotomy. Resection via a laparoscope will not be acceptable.

5.12 Intraoperative Findings which Prevent Implementation of Protocol Treatment

Patients with isolated, distant, or noncontiguous intra-abdominal metastases at the time of operation (Dukes' D), **even if resected**, are ineligible to receive the designated protocol treatment; they will be followed for survival information. Such extent of intra-abdominal disease must be confirmed histologically. Any suspicious extra-colonic lesion seen at operation should be biopsied. Patients with tumors which require opening of the pelvic peritoneum in order to define the extent of tumor will be considered to have rectal tumors, and are not eligible.

5.13 Pathologic Findings which Prevent Further Chemotherapy

Upon receipt of the final surgical pathology report from the contributing institution's pathology department, Dukes' A, B1, and D patients will have perioperative 5-FU ***discontinued***, if so assigned, and will receive NO further adjuvant treatment. These patients will be treated at the discretion of their physician and survival data will continue to be collected on these patients until the time of death. Dukes' B2 patients will have perioperative 5-FU ***continued*** to completion, if so assigned, but will receive NO further adjuvant treatment. These patients will be followed on observation until relapse or death.

5.2 Chemotherapy Guidelines

5.21 Perioperative 5-FU

Patients randomized to the perioperative 5-FU arm must begin treatment within 24 hours of the completion of the curative resection. They will receive 600 mg/m^2/day of 5-FU as a continuous IV infusion over 24 hours for 7 days. Doses of 5-FU should be based on actual weight. See Section 5.41.

Upon receipt of the final surgical pathology report from the contributing institution's pathology department, Dukes' A, B1, and D patient should have perioperative 5-FU chemotherapy ***discontinued***. Dukes' B2, B3, and C patients should have perioperative 5-FU ***continued*** to completion.

Rev. 9/97 5.22 Adjuvant 5-FU and Leucovorin

A cycle of therapy will consist of 5 consecutive days of chemotherapy. The full course of adjuvant therapy will consist of 6 cycles.

Agent	Dose (per day)	Route	Schedule	Instructions
Leucovorin	20 mg/m²	IV push	days 1-5	
5-FU	425 mg/m²	IV push	days 1-5	Give immediately after Leucovorin

Dose should be based on actual weight (estimated dry weight if patient has edema).

5.221 Cycles will be repeated at the end of 4 weeks (day 29), 8 weeks (day 57) and then every 4 weeks for a total of 6 cycles.†

Day On Study	1	29	57	85	113	141	STOP
Treatment (Days 1-5)	*	*	*	*	*	*	

† Initiation of subsequent treatment cycles may be delayed up to 4 days if necessary for logistical reasons.

5.3 Adverse Reaction Reporting Requirements

Rev. 3/10 **The descriptions and grading scales found in the revised NCI Common Terminology Criteria for Adverse Events (CTCAE) version 4.0 will be utilized for AE reporting. All appropriate treatment areas should have access to a copy of the CTCAE version 4.0. A copy of the CTCAE version 4.0 can be downloaded from the CTEP web site (http://ctep.cancer.gov).**

5.31 This protocol does not contain IND agents; toxicities occurring on this treatment should be considered commercial.

5.32 The following adverse reactions must be reported to ECOG and NCI in the manner
Rev. 9/97 described in the table below. In addition your local IRB should be notified.

NOTE: CALGB participants should follow ECOG guidelines regarding ADR reporting procedures.

Rev. 3/95 **NOTE:** NSABP participants should follow ECOG guidelines regarding ADR reporting procedures. In addition, a copy of the ADR Form should be forwarded to the NSABP Biostatistical Center.

NSABP Biostatistical Center Mailing Address:
NSABP Biostatistical Center
230 McKee Place
Suite 600
Pittsburgh, Pennsylvania 15213

For All Drug Arms:

Rev. 8/99

	Grades 4- 5, unexpected[1]	Death due to Rx or within 30 days of Rx[2]
ECOG ADR Form to NCI within 10 days	X	X
ECOG ADR Form to ECOG DM Office within 10 days	X	X
Notify local IRB within 10 days	X	X

1 Any unexpected toxicity not reported in the literature or the package insert must be reported, as well as any increased incidence of a known ADR reported in the package insert or literature.

2 Any death from any cause while a patient is receiving treatment on this protocol or up to 30 days after the last dose of protocol treatment, or any death which occurs more than 30 days after protocol treatment has ended, but is clearly felt to be treatment related, must be reported.

Rev. 9/97
Rev. 6/94, 9/95
Rev. 3/95, 8/99

NCI Telephone Number: (301) 230-2330
NCI FAX Number: (301) 230-0159
NCI Mailing Address:
IDB
P.O. Box 30012
Bethesda, MD 20824

ECOG Telephone Number: (617) 632-3610
ECOG Fax Number: (617) 632-2990
ECOG Mailing Address:
ECOG Coordinating Center
ATTN: ADR
Frontier Science
303 Boylston Street
Brookline, MA 02445-7648

Rev. 6/94

ECOG requires ADRs to be reported on the Adverse Reaction (ADR) Form For Investigational Drugs (#391RF). The form must be signed by the treating investigator.

5.33 Non-Treatment Related Toxicities

Rev. 9/95, 9/97

Toxicities which fall within the definitions listed above must be reported as an ADR/second primary regardless if they are felt to be treatment related or not. Toxicities unrelated to treatment that do NOT fall within the definitions above, must simply be clearly documented on the ECOG flow sheets which are submitted to the ECOG Coordinating Center (ATTN: DATA) according to the Records to be Kept Section (11.0).

5.34 All Southwest Oncology Group (SWOG) investigators are responsible for reporting of adverse drug reactions according to the NCI and Southwest Oncology Group Guidelines.

SWOG investigators must:

- Call the SWOG Operations Office at (210) 677-8808 within 24 hours of any suspected adverse event deemed either drug-related, or possibly drug-related.

  Instructions will be given as to the necessary steps to take depending on whether the reaction was previously reported, the grade (severity) of the reaction, study phase, and whether the reaction was caused by investigational and/or commercial agent(s). The SWOG Operations Office will immediately notify ECOG.

The following ADRs attributed to commercial agent(s) should be reported to the Investigational Drug Branch, Cancer Therapy Evaluation Program, within 10 working days:

- Any ADR that is both serious (life threatening, fatal) and unexpected.
- Any increased incidence of a known ADR that has been reported in the package insert or the literature.
- Any death on study if clearly related to the commerical agent(s).

Rev. 6/94 The adverse reaction must be documented on the ECOG ADR Form (#391RF) and a copy mailed to:

Investigational Drug Branch
P.O. Box 30012
Bethesda, Maryland 20824

In addition, within 10 days the investigator must send:

- the original of the above ADR report
- all data records for the period covering prestudy through the adverse event
- documentation of IRB notification, to the following address:

ADR Program
SWOG Operations Office
14980 Omicron Drive
San Antonio, TX 78245-3217

At the Operations Office, a multilayered review will be performed and any pertinent findings will be forwarded to ECOG, the NCI Study Coordinator, and Statistical Center, along with any supporting documentation. The original should be sent whenever forwarding an ADR Form to ECOG.

Rev. 8/99 5.35 The following adverse reactions must be reported to ACOSOG, the NCI, and your local IRB in the manner described below:

For all Drug Arms:

	**Grade 4-5 unexpected**	**Death due to Rx or within 30 days of Rx**
ADR Form 391 RF to NCI within 10 days	X	X
ADR Form 391 RF to ACOSOG Coordinating Center within 10 days	X	X
Notify local IRB within 10 days	X	X

1 Any unexpected toxicity not reported in the literature or the package insert must be reported.
2 Any death from any cause while a patient is receiving treatment on this protocol or up to 30 days after the last dose of protocol treatment, or any death which occurs more than 30 days after protocol treatment has ended but which is felt to be treatment related, must be reported.

All ADRs should be reported on the Adverse Reaction (ADR) Form for Investigational Drugs (#391RF). The form must be signed by the treating investigator and faxed to the ACOSOG Coordinating Center at **(312) 202-5710** or **(312) 202-5711**. A copy should be sent to NCI.

NCI Telephone Number: (301) 230-2330
NCI Fax Number: (301) 230-0159
NCI Mailing Address:
Investigational Drug Branch
P.O. Box 30012
Bethesda, MD 20824

Rev. 8/99 5.36 Non-Treatment Related Toxicities

Toxicities which fall within the definitions listed above must be reported as an ADR/Second primary regardless if they are felt to be treatment related or not. Toxicities unrelated to treatment that do NOT fall within the definitions above must simply be clearly documented on the ECOG Toxicity Form and faxed to the ACOSOG Coordinating Center.

5.4 Dose Modifications

Rev. 3/10 **NOTE:** Dose modifications are defined using CTC Version 2 terminology (see Appendix II).

5.41 Drug Toxicity and Cessation of Perioperative 5-FU Therapy *****

No dose modification will be implemented; infusions will be discontinued if any of the following reactions are apparent:

Rev. 8/99
(a) Leukocytopenia (< 3000/mm^3)*
(b) Thrombocytopenia (< 100,000/mm^3)*****
(c) An intervening significant postoperative complication
(d) If patient develops angina, thought to be secondary to 5-FU, the infusion should be discontinued.
(e) Grade II mucositis

Rev. 8/99 ***** WBC, PLT will be checked on post-operative day 2 and day 5.

Rev. 9/97 5.42 Dose Modifications During Treatment with 5-FU + Low-Dose Leucovorin

Rev. 3/10 **NOTE:** Dose modifications are defined using CTC Version 2 terminology (see Appendix II).

If multiple toxicities are seen, the dose administered should be based on the most severe toxicity experienced. Dosage reductions apply to the dose of chemotherapy given on the preceding treatment cycle, and should be based on toxicities observed since the previous dose of chemotherapy. (The dose of leucovorin is not modified for chemotherapy toxicity.)

5.421 Toxicity Based on Interval Toxicity

Toxicity	Percent of Dose Resulting In Toxicity 5-FU	Leucovorin
Hematology Nadirs (/mm³)		
WBC 1000-2500	80%	100%
WBC < 1000	70%	100%
PLT 25,000-75,000	80%	100%
PLT < 25,000	70%	100%
Diarrhea		
CTC Grade 1	100%	100%
CTC Grade 2	80%	100%
CTC Grade 3-4	70%	100%
Stomatitis		
CTC Grade 1	100%	100%
CTC Grade 2	80%	100%
CTC Grade 3-4	70%	100%

IF WBC < 3,500/mm³ and or platelets < 100,000/mm³ at the start of a treatment cycle, hold therapy and repeat counts weekly x 2. If the blood counts are still below these levels, adjuvant therapy should be discontinued.

The dose of 5-FU may be re-escalated only in patients experiencing Grade 1 diarrhea or mucositis and for hematologic toxicity, provided they have not had toxicity based on the most prior treatment. The drug should be re-escalated to the next highest dose level.

Rev. 9/97

5.5 Supportive Care

Any antiemetic regimen can be used at the discretion of the investigator.

Rev. 9/97 5.51 Mucositis has been a common toxicity in patients receiving 5-day courses of 5-FU and Leucovorin. Preliminary reports suggest that oral cryotherapy may decrease this toxicity (24). Patients who are assigned to this arm may be offered this therapy; if performed, flow sheets should clearly indicate the patient was so treated.

Method: 5 minutes prior to each dose of 5-FU and Leucovorin patients will place ice chips in their mouths and will swish the ice continuously around their oral cavities for 30 minutes, replenishing the ice chips as needed. Patients with dentures should remove them prior to treatment.

Rev. 9/97 5.52 Supportive Care Options During Therapy with 5-FU + Leucovorin

Diarrhea has been a common toxicity with 5-FU + Leucovorin therapy, frequently requiring parenteral hydration and significant dose delays and modifications. Preliminary reports suggest that somatostatin analogue (Sandostatin) may more rapidly and completely control diarrhea, which may lead to better protocol
Rev. 9/97 compliance and safety (25).

Method: In patients with ≥ Grade 1 diarrhea, chemotherapy should be modified as appropriate by protocol. In addition, patients who have ≥ Grade 2 diarrhea should be considered for Sandostatin therapy, 50-100 ug SQ every 8 hours for up to 3 days. When utilized, Sandostatin therapy and response should be recorded on flow sheets.

Rev. 9/97, 8/99 ECOG investigators may wish to consider entering patients with at least Grade 2 chemotherapy-related diarrhea on E1295, *Randomized Trial of High-Dose Versus Conventional Dose Octreotide Acetate Versus Loperamide In The Treatment of Chemotherapy-Related Diarrhea In Patients With Colorectal Cancer*.

Appropriate dose modifications should continue to be implemented in subsequent treatments, based on the highest grade of diarrhea experienced.

5.6 Duration of Therapy

5.61 Perioperative 5-FU

Upon receipt of the final surgical pathology report from the contributing institution's pathology department, Dukes' A, B1, and D patient should have perioperative 5-FU chemotherapy ***discontinued***. These patients will be removed from the study, and survival data will be collected on them until death. Dukes' B2, B3, and C patients should have perioperative 5-FU ***continued*** to completion.

5.62 Adjuvant 5-FU + Leucovorin

Rev. 9/97 Patients receiving adjuvant 5-FU/leucovorin continue the therapy for a total of 6 months or until recurrence. Once a patient has recurred according to the criteria in Section 6.1, he/she is considered off study and will be followed for survival.

6.0 MEASUREMENT OF EFFECT

Note: Biopsy confirmation of disease recurrence should be obtained whenever possible with acceptable risk.

6.1 Criteria for Disease Progression

6.11 Local recurrence of malignant disease proven by biopsy.

6.12 Positive liver scan, CT, or MRI. This observation should be confirmed by repeat determinations, 1 month apart.

6.13 Pulmonary metastasis by chest x-ray or CT scan.

6.14 Bony metastasis by routine x-ray, if previous x-ray of suspected lesion was normal at onset of study. Abnormal bone scans have to be confirmed by routine x-ray and/or biopsy.

6.15 A persistent rise in CEA titer of 10 times upper normal value, confirmed on 2 separate determinations at 1 month intervals, in patients who had an initially normal CEA value upon entry into the study. The determination should be performed by the same laboratory, using the same method. The method used should be specified on the Serum Marker Form. Every effort should be made to document the tumor recurrence, including exploratory laparotomy if necessary.

6.16 Ascites or pleural effusion with positive cytology for malignant cells.

6.17 Anastomotic recurrence, proven by biopsy.

6.18 Intraabdominal, abdominal wall or incisional recurrence proven by biopsy.

6.2 The following are not acceptable as evidence of progression:

6.21 Ascites with negative cytology for tumor cells.

6.22 Pleural effusion with negative cytology.

6.23 Computerized Axial Tomography (CAT), MRI, or liver scan findings alone on a single determination.

6.24 Bone scan alone.

6.25 Barium enema findings alone.

6.26 "Blumer" shelf.

7.0 STUDY PARAMETERS

7.1 Before Randomization

All Patients:

Rev. 6/94 a) CXR must be done ≤ 2 weeks prior to resection or randomization.
b) All initial lab values must be done ≤ 2 weeks prior to randomization.
Rev. 8/99 c) If white blood cell count (WBC) & platelet count are abnormal, they must be repeated within 48 hours prior to randomization.
d) CT scan or MRI is suggested, but optional.

NOTE: When documenting initial results on the ECOG Flow Sheet, please clearly record the actual date on which the scan/test was done. Do NOT use the date of Day 1 of protocol treatment unless the scan/test was actually done that day.
Rev. 8/99 All follow-up WBC and platelet counts should be done < 48 hours prior to the day of treatment.

		Initial	1 Month Post-Op	Year 1 (Dukes' B2, B3, C)	Year 2 (Dukes' B2, B3, C)	Year 3, 4 (Dukes' B2, B3, C)	Year 5 & After (Dukes' B2, B3, C)
Rev. 9/97	Eval. for Surgical Complications		X				
	History & Physical Examination	X	X	q 3 mo	q 3 mo	q 6 mo	q 12 mo
	Height & Weight	X	X	q 3 mo	q 3 mo	q 6 mo	q 12 mo
	ECOG Performance Status	X	X	q 3 mo	q 3 mo	q 6 mo	q 12 mo
Rev. 8/99	WBC & Platelet Count[1]	X	X	q 3 mo	q 6 mo	q 12 mo	q 12 mo
	Creatinine & Bilirubin	X	X	q 3 mo	q 6 mo	q 12 mo	q 12 mo
	CEA [2]	X	X	q 3 mo	q 3 mo	q 6 mo	q 12 mo
	Chest X-Ray	X		q 6 mo	q 6 mo	q 12 mo	q 12 mo
Rev. 3/95 Rev. 12/08	Colonoscopy or Barium Enema[3]	X		q 12 mo	q 12 mo	q 24 mo	q 3-5 yrs [6]
	Pregnancy Test[4]	X					
Rev. 9/97							
	Submission of Slides & Paraffin-[5] Embedded Tumor & Normal Tissue		X				

Rev. 7/96 [1] Should be done Day 2 and Day 5 after surgery for patients receiving perioperative chemotherapy.
Rev. 3/95, 8/99 [2] Optional.
Rev. 9/97 [3] Should be done ≤ 4 weeks prior to randomization/resection.
[4] For female patients of childbearing potential who will be receiving chemotherapy.
[5] MUST be submitted for ECOG patients within 1 month of surgery **(see Section 10.0 for details)**. CALGB patients registering to
Rev. 3/95 E3293 must submit only 1 paraffin block from primary tumor.
Rev. 3/95 6 It is recommended that patients receive a Colonoscopy or Barium Enema every 3-5 years.
Rev. 7/96,9/97
Rev. 12/08

7.2 After Pathologic Staging:

Dukes' B3 and C Patients Only:

Rev. 8/99
Rev. 9/97 a) WBC, platelet count, creatinine, and bilirubin should be done ≤ 1 week prior to beginning adjuvant chemotherapy (5-FU/leucovorin).

Rev. 8/99 b) **WBC and platelet count should be done weekly while patients are receiving adjuvant chemotherapy**

Rev. 9/97, 8/99

(5-FU/leucovorin). At completion of chemotherapy, the schedule above for WBC and platelet count should be followed.

8.0 DRUG FORMULATION AND PROCUREMENT

8.1 **5-Fluorouracil** (Fluorouracil, 5-FU, Adrucil, Efudex)

8.11 Classification and Description

Classification: Antimetabolite

Description: 5-FU is a commercially available injection. It is colorless to faint yellow, with a pH of 9.2 (adjusted with sodium hydroxide).

8.12 Mode of Action

Fluorouracil is a pyrimidine antagonist that interferes with nucleic acid biosynthesis. The deoxyribonucleotide of the drug inhibits thymidylate synthetase, thus inhibiting the formation of thymidylic acid from deoxyuridylic acid, thus interfering in the synthesis of DNA. It also interferes with RNA synthesis.

8.13 Storage and Stability

Stable for prolonged periods of time at room temperature if protected from light. Inspect for precipitate; if apparent, agitate vial vigorously or gently heat to not greater than 140˚ F in a water bath. Do not allow to freeze.

8.14 Dose Specifics

See Section 5.2 regarding specifics of dose delivery for this protocol.

8.15 Administration

The drug may be given IV push, IV continuous infusion, arterial infusion, intracavitary, intraperitoneally, topically, or orally mixed in water, grape juice, carbonated beverage.

See Section 5.2 for specifics of administration in this protocol.

8.16 Incompatibilities

Incompatible with Doxorubicin and other anthracyclines.

8.17 Availability

Commercially available in 500 mg/10 ml ampules and vials, and 1 gm/20 ml, 2.5 gm/50 ml, and 5 gm/100 ml vials.

8.18 Side Effects

1. Hematologic: Leukopenia, thrombocytopenia, anemia.
2. Dermatologic: Dermatitis, nail changes, hyperpigmentation, hand-foot syndrome with protracted infusions, alopecia. Exposure to strong sunlight may intensify skin reactions to the drug.

3. Gastrointestinal: Nausea, vomiting, anorexia; diarrhea; stomatitis, mucositis, more common with 5-day infusion, occasionally dose-limiting.

4. Neurologic: Cerebellar syndrome (headache and cerebellar ataxia), disorientation, confusion, euphoria, ataxia, and nystagmus.

5. Cardiac: Angina, noted with continuous infusion.

6. Ophthalmic: Eye irritation, nasal discharge, watering of eyes, blurred vision, and photophobia.

7. Other: Weakness and malaise.

8.19 Patient Care Implications

1. Monitor blood counts.

2. Administer antiemetics as indicated.

3. Monitor for diarrhea. Encourage fluids and treat symptomatically.

4. Assess for stomatitis - oral care recommendations as indicated.

5. Monitor for neurologic symptoms (headache, ataxia).

6. Patients on continuous infusions may need instruction regarding central IV catheters and portable IV or IA infusion devices.

7. Inform patient of potential alopecia.

8.110 References

Hansen R., Quebbeman E., Ausman R., et al. (1989). Continuous Systemic 5-Fluorouracil in Advanced Colorectal Cancer: Results in 91 patients. *Journal of Surgical Oncology, 40*,177-181.

Freeman N.J., Costanza M.E. (1988). 5-Fluorouracil-Associated Cardiotoxicity. *Cancer 61*, 36-45.

Rev. 9/97 8.2 Leucovorin Calcium

8.21 Other Names

Leucovorin, Wellcovorin, citrovorum factor, folinic acid, 5-formyl tetrahydrofolate, LV, LCV.

8.22 Classification

Tetrahydrofolic acid derivative.

8.23 Mode of Action

Leucovorin acts as a biochemical cofactor for 1-carbon transfer reactions in the synthesis of purines and pyrimidines. Leucovorin does not require the enzyme dihydrofolate reductase (DHFR) for conversion to tetrahydrofolic acid. The effects of methotrexate and other DHFR-antagonists are inhibited by leucovorin.

Leucovorin can potentiate the cytotoxic effects of fluorinated pyrimidines (i.e., fluorouracil and floxuridine). After 5-FU is activated within the cell, it is accompanied by a folate cofactor, and inhibits the enzyme thymidylate synthetase, thus inhibiting pyrimidine synthesis. Leucovorin increases the folate pool, thereby increasing the binding of folate cofactor and active 5-FU with thymidylate synthetase.

8.24 Storage and Stability

All dosage forms are stored at room temperature. The reconstituted parenteral solution, 10 mg/ml, is stable for at least 7 days at room temperature. At concentrations of 0.5-0.9 mg/ml the drug is chemically stable for at least 24 hours at room temperature under normal laboratory light. The oral solution, 1 mg/ml, is stable for 14 days refrigerated and 7 days at room temperature.

8.25 Dose Specifics

Leucovorin will be given at a dose of 20 mg/m^{2}IV push, days 1-5. Doses should be based on actual weight (estimated dry weight if patient has edema). A cycle of therapy consists of 5 consecutive days of chemotherapy. The full course of adjuvant therapy will consist of 6 cycles.

Cycles will be repeated at the end of 4 weeks (day 29), 8 weeks (day 57), and then every 4 weeks for a total of 6 cycles.

8.26 Preparation

The 50 and 100 mg vials for injection are reconstituted with 5 and 10 ml of sterile water or bacteriostatic water, respectively, resulting in a 10 mg/ml solution. The 350 mg vial is reconstituted with 17 ml of sterile water resulting in a 20 mg/ml solution. The 60 mg bottle for oral solution is reconstituted with 60 ml of aromatic elixir provided, resulting in a 1 mg/ml oral solution.

8.27 Administration

IV push.

8.28 Compatibilities

Leucovorin (0.5-0.9 mg/ml) is chemically stable for at least 24 hours in normal saline, 5% dextrose, 10% dextrose, Ringer's injection or lactated Ringer's injection. Leucovorin (0.03, 0.24 and 0.96 mg/ml) is stable for 48 hours at room and refrigeration temperatures when admixed with floxuridine (FUDR, 1, 2 and 4 mg/ml) in normal saline. Leucovorin is also compatible with fluorouracil.

8.29 Availability

Commercially available.

8.210 Side Effects

Rev. 4/98

1. Dermatologic: Skin rash.
2. Gastrointestinal: Nausea, upset stomach, diarrhea.
3. Allergic: Skin rash, hives, pruritus.
4. Pulmonary: Wheezing (possibly allergic in origin).
5. Other: Headache; may potentiate the toxic effects of fluoropyrimidine therapy, resulting in increased hematologic and gastrointestinal (diarrhea, stomatitis) adverse effects.

8.211 Nursing Implications

1. Observe for sensitization reactions.
2. When given with fluoropyrimidines monitor closely for diarrhea and stomatitis.

8.212 References

Arbuck SG. Overview of clinical trials using 5-fluorouracil and leucovorin for the treatment of colorectal cancer. Cancer 1989; 63: 1036-1044.

Bleyer WA. New vistas for leucovorin in cancer chemotherapy. Cancer 1989; 63: 995-1007.

Grem JL. 5-Fluorouracil plus leucovorin in cancer therapy. In: Cancer: Practices and Principles of Oncology Updates. Devita VT, Hellman S, Rosenberg SA, editors. 1988; 2(7): 1-12.

9.0 STATISTICAL CONSIDERATIONS

Rev. 9/97 The primary objective of this study is to compare 6 months of adjuvant therapy with 5-FU and leucovorin to the same adjuvant therapy enhanced with perioperative therapy with continuous infusion
Rev. 4/98 5-FU in Dukes' B3 and C patients with regard to the disease free interval and survival. This study is designed to be able to detect a clinically meaningful improvement in survival with 82% power while maintaining an overall significance level of 5% in a one-sided test, assuming
Rev. 9/95 patients are followed for 3 years after closure of the study to accrual. If 5 years of follow-up time were available, the power will be 91%. For Dukes' C patients, a clinically meaningful improvement in survival was taken to be an improvement in 5-year survival from 55% to 65%. For Dukes' B3 patients, this is an improvement in 5-year rates from 75% to 81%. To estimate the required sample sizes we assume exponential survival and a group sequential designed with an O'Brien-Fleming upper bound for early rejection of the null hypothesis of no treatment difference.

Assuming a 5-year survival rate of 75% for Dukes' B3 patients and 55% for Dukes' C patients in the adjuvant therapy alone arm, this study will require 800 analyzable patients (400 in each arm) to obtain the stated power using a stratified logrank test. Calculations assume that approximately 70% of the Dukes' B3 and C patients will be Dukes' C and 30% will be Dukes' B3. Since pathological staging is determined only after randomization, the accrual goal needs to be expressed in terms of Dukes' A,

B, C, and D patients. Using data on staging* it is projected that 40% of all patients will be Dukes' B3 or C thus requiring a total accrual goal of 2000 patients to obtain the required number of B3 and C patients. As these projections are very approximate, accrual will be closely monitored during the first year and the accrual goals will be revised as necessary. For Dukes' A, B1, B2, and D patients, there will be no formal treatment comparisons. However, these patients will be followed to gather more information about disease progression and survival.

Perioperative therapy will require the full participation of the group's surgeons and will be feasible only in member institutions. Thus, this study will not benefit from referrals from outside institutions, which normally comprise 60-75% of the patient population in adjuvant trials. Assuming that the annual rate will be 750 - 875 total patients/year (300-350 B3 and C patients / year), < 25% of the current accrual rate to intergroup study E2288, this study will need about 2-3 years to complete accrual.

We will assume that the patients will be followed for 3 years after the closure of the study to accrual and that 3 interim analyses will be conducted at 2.5 years (1/2 year before the end of accrual), 3.5 years, and 5 years. If the study is not terminated at the end of 5 years, a final analysis will be conducted at 6 years (3 years of follow-up after closure to accrual). The critical values corresponding to these times will be 3.3358, 2.4524, 1.8978, and 1.7480 corresponding to nominal significance levels of .0004, .007, .03, and .04. The chosen intermin analysis times closely approximate equal increments in the total number of expected deaths (25%, 50%, and 75% respectively).

---

* DeVita, Vincent T., Hellman, Samuel, & Rosenberg, Steven A. (1985). *Cancer: Principles & Practice of Oncology*, (Vol. 1, 2nd ed.). Philadelphia: J.B. Lippincott Company.

10.0 PATHOLOGY REVIEW

Rev. 3/95, 7/96, 8/99

NOTE: The submission of pathology material is required for ECOG patients and encouraged for CALGB patients entered on this protocol. Submission of tissue samples is not required for SWOG, ACOSOG, EPP or NSABP patients.

Rev. 11/93, 7/96

When randomizing ECOG and CALGB patients to E1292/C9395, it is strongly recommended that they be registered to laboratory companion protocol E3293.

E3293 will involve molecular genetic analysis of allelic deletions involving the p53 gene on chromosome 17p and the DCC gene on chromosome 18q. These proposed prognostic markers will be evaluated by survival analysis. DNA will be derived from cryostat sections. Deletions will be determined by restriction fragment length polymorphism as in previous studies using probes for 17p and 18q.

10.1 Justification

Rev. 9/97

Tumor tissue and normal colonic tissue are being requested for tissue banking and future lab analysis. Paraffin blocks will be stored at the ECOG Pathology Coordinating Office.

10.2 Pathology Material to be Submitted

Rev. 7/96

10.21 Paraffin/Slide Samples (ECOG institutions only)

Rev. 3/95

NOTE: Submission of paraffin blocks and slides is mandatory for ECOG Patients.

10.211 Patient Information Form (see Appendix III).

10.212 Surgical pathology report.

10.213 At least one (1) formalin-fixed, paraffin-embedded block from a representative area of the primary tumor.

10.214 One (1) formalin-fixed, paraffin-embedded block from normal colonic mucosa.

10.215 1 H & E stained slide from each block of tumor or normal colonic mucosa submitted.

(Any extra tumor blocks would be appreciated by Dr. Hamilton and the ECOG Central Tissue Repository).

Rev. 7/96 10.22 Frozen Tissue Samples (ECOG Institutions only)

Rev. 9/97 Frozen tissue samples are no longer being collected.

Rev. 7/96 10.23 CALGB Institutions

The following should be submitted to:

CALGB Pathology Office
Maurice Barcos, M.D., Ph.D.
Roswell Park Cancer Institute
Department of Pathology
Elm and Carlton Streets
Buffalo, NY 14263
Tel: 716-845-4443
Fax: 716-845-8077

1. One paraffin embedded block with REPRESENTATIVE TUMOR properly identifying:

   a. Patient's name
   b. CALGB patient number and ECOG patient number
   c. CALGB study number and ECOG study number

2. Original Patient Information Form (H&E Section to be completed by CALGB Pathology Office)

3. Original completed CALGB Form C-350

4. A copy of the responsible pathologist's surgical and pathology report from the TREATING institution, and, if applicable. the REFERRING institution.

These tissue blocks will be sectioned by the CALGB Pathology Office, according to ECOG Pathology Coordinating Office specifications, and then sent to the ECOG PCO, along with the completed Patient Information Form.

Rev. 9/97

10.3 Tissue Processing and Distribution:

Rev. 9/95 At the time of randomization, the ECOG Coordinating Center will contact the Research Coordinator (Mrs. Rahj Robinson) in the Gastrointestinal Pathology Unit at Johns Hopkins.

Rev. 9/97

At the time of contact with the pathologist at the contributing institution, the Research Coordinator will also encourage the pathologist to embed extra blocks of formalin-fixed tumor for routine histology. **These blocks will be used for immuno-histochemistry and flow cytometry. The blocks will then be kept at the ECOG Pathology Coordinating Office's Central Tissue Repository for use in future studies.**

10.31 Paraffin/Slide Samples

As much of the primary tumor and normal colonic mucosa as possible should be formalin-fixed and embedded for histological examination. At least one representative paraffin block from the primary tumor, one paraffin block from normal colonic mucosa, 1 H&E stained recut slide from each block, the surgical pathology report, and the Patient Information Sheet (see Appendix III), should be sent to the ECOG Pathology Coordinating Office (PCO) for handling:

Rev. 3/95, 9/95, 9/97

ECOG Pathology Coordinating Office
Evanston Hospital- Room B624
2650 Ridge Avenue
Evanston, IL 60201-1797

These samples will be distributed from the PCO to Dr. Hamilton's laboratory when patients are registered to E3293. **Extra blocks will be returned to the ECOG Pathology Coordinating Office's Central Tissue Repository for use in future studies.**

Rev. 7/96

CALGB Institutions:

CALGB institutions should submit one parraffin embedded block from a representative area of the primary tumor to the CALGB Pathology Office (See Section 10.23). The CALGB Pathology Office will then prepare an H&E stained slide from the block, and send these to the ECOG Pathology Coordinating Office. CALGB institutions are not responsible for fresh frozen tissue or normal colonic mucosa.

11.0 RECORDS TO BE KEPT

Rev. 9/95
Rev. 8/99
The following forms must be submitted to the ECOG Coordinating Center, Frontier Science, 303 Boylston Street, Brookline, MA 02445 (ATTN: DATA).

	Form	To Be Submitted
Rev. 7/96	Copy of completed ECOG checklist	Within one week of registration/re-registration (Non-ECOG participants)
	* ECOG Colon Surgical Adjuvant On-Study Form (#559)	Within two weeks of surgery
	* Operative Report	Within two weeks of surgery
	* Pathology Report	Within two weeks of surgery
Rev. 9/97	* ECOG CTC Flow Sheet (#466)	Every 3 months while on treatment and at each follow-up in accordance with the Study Parameters schedule.
	ECOG Follow-Up Form (#464): Parts A, B, C, D, E	Every 3 months while on treatment and at completion of treatment
	***** Parts A, B	Off treatment:<br>• Every 3 months if patient is < 2 years from study entry<br>• Every 6 months if patient is 2 - 4 years from study entry<br>• Every 12 months if patient is > 5 years from study entry
	* ECOG Serum Marker Form (Revised) (#294R)	Submit forms as defined in Section 7.0
Rev. 6/94	Adverse Drug Reaction Report Form for Investigational Drugs (#391RF)	Within 10 days of reportable toxic event as defined in Section 5.3.

* These forms are to be submitted for all canceled patients according to the above schedule.

NOTE: CALGB participants should follow ECOG guidelines regarding Data Form submission procedures.

11.1 SWOG Forms Submission:

The original data forms as listed above should be submitted at the required intervals to the following address:

Southwest Oncology Group Statistical Center
Fred Hutchinson Cancer Research Center
1124 Columbia Street, MP-557
Seattle, WA 98104-2092

Include the ECOG protocol number and patient sequence number as well as the Southwest Oncology Group study number and patient number. It is not necessary to submit extra copies.

Rev. 3/95
11.2 NSABP Forms Submission

The original data forms as listed above should be submitted at their required intervals to the NSABP Biostatistical Center at the following address:

NSABP Biostatistical Center
Suite 600
230 McKee Place
Pittsburgh, PA 15213

Rev. 8/99 11.3 ACOSOG Forms Submission

The ACOSOG Coordinating Center will provide, to the institutions, **DataFax Shuttle Forms** (barcoded forms) for **each** of the data forms listed in Section 11.0. These Shuttle Forms, as well as the ECOG Case Report Forms, will be available to institutions through the ACOSOG web page (http://www.acosog.org) as PDF files. The ECOG forms should be attached to the appropriate ACOSOG Shuttle Forms and **faxed in** at the required intervals to the ACOSOG Coordinating Center at **(312) 202-5710** or **(312) 202-5711** (see SOP:DataSub for additional information on forms access and submission). The ACOSOG Coordinating Center will forward the CRFs in the form of fax images to the ECOG Statistical Office.

Include on each form the ECOG protocol number (E1292) and patient number, as well as the ACOSOG protocol number and patient number.

12.0 PATIENT CONSENT AND PEER JUDGMENT

All institutional, NCI, FDA, state and federal regulations concerning informed consent and peer judgment will be fulfilled.

Rev. 6/94

13.0 REFERENCES

1. DeVita V.T., Hellman S., Rosenberg S.A. (1989). Colorectal cancer. *Cancer Principles & Practice of Oncology; Third Edition*, J.B. Lippincott Company, 895-952.

2. Fisher B.: NSABP Protocol C-02.

3. Taylor I., *et al.* (1979). Adjuvant cytotoxic liver perfusion for colorectal cancer. *British Journal of Surgery, 66*, 833.

4. Progress Report NSABP. (April, 1992). 379 - 384.

5. Fielding P.L., Hittinger R., Grace R.H., *et.al.* (1992). Randomized controlled trial of adjuvant chemotherapy by portal-vein perfusion after curative resection for colorectal adenocarcinoma. *The Lancet , 340*, 502-506.

6. O'Connell M., Moertel C., Wieand H., *et al.* (1989) Biomedical modulation of fluorouracil: evidence of significant improvement of survival and quality of life in patients with advanced colorectal carcinoma. *Journal of Clinical Oncology , 7*, 1407-1417.

7. Moertel C.G., Fleming T.R., Macdonald J.S., *et al.* (1990). Levamisole and fluorouracil for adjuvant therapy of resected colon carcinoma. *New England Journal of Medicine, 322*, 352-358.

8. Moertel C.G., Fleming T.R., Macdonald J.S., *et al.* (1992). The intergroup study of fluorouracil (5-FU) plus levamisole (LEV) and levamisole alone as adjuvant therapy for Stage C colon cancer. A final report. *ASCO Proceedings AA*, 457.

9. Schiller J.H., Witt P.L. (1992). Levamisole: clinical and biological effects. In DeVita V.T., Hellman S., Rosenburg S.A., *Biologic Therapy of Cancer, 2:9.* J.B. Lippincott Company, 1-14.

10. Gray B., deZwart J., Fisher R., *et al.* (1987). The Australia and New Zealand trial of adjuvant chemotherapy in colon cancer. *Adjuvant Therapy of Cancer V.* Grune & Stratton, 537-546.

Rev. 9/97 11. Kemeny, M.M., Ibrahim, J., Benson, A.B. III (1997). Postoperative complications of continuous infusion 5-FU following curative resection of colon cancer: ECOG 1292. *Proc. ASCO, 16: 260a.*

Rev. 9/97 12. Haller D, Catalano P, Macdonald JS, Mayer RJ (1996) Fluorouracil (FU), Leucovorin (LV) and Levamisole (LEV) Adjuvant Therapy for Colon Cancer: Preliminary Results of INT-0089. ASCO Proceedings.

Rev. 9/97 13. Haller D, *et al.* (1997) Fluorouracil (FU), Leucovorin (LV) and Levamisole (LEV) Adjuvant Therapy for Colon Cancer: Four-year Results of INT-0089. ASCO Proceedings.

14. Laurent-Puig P, Olschwang S, Dalattre O, et al. (1992). Survival and acquired genetic alterations in colorectal cancer. Gastroenterology 102:1136-41.

15. Hamilton SR. (1992). Molecular genetic alterations as potential prognostic indicators in colorectal carcinoma. *Cancer,* 69: 1589-91.

16. Fisher, B. et al. (1988) Postoperative adjuvant chemotherapy or radiation therapy for rectal cancer: Results from NSABP protocol R-01. *Journal of the National Cancer Institute,* 80:21-29.

17. Wolmark, N. et al. (1988) Postoperative adjuvant chemotherapy or BCG for colon cancer: Results from NSABP protocol C-01. *Journal of the National Cancer Institute,* 80:30-36.

18. Laurie, J. A. et al. (1989) Surgical adjuvant therapy of large-bowel carcinoma: An evaluation of levamisole and the combination of levamisol and flourouracil. *Journal of Clinical Oncology,* 7:1447-1456.

19. Milano, G. et al. (1992) Influence of sex and age on flourouracil clearance. *Journal of Clinical Oncology,* 10:1171-1175.

20. Gastrointestinal Tumor Study Group. (1984) Adjuvant therapy of colon cancer: Results of a prospectively randomized trial. *New England Journal of Medicine,* 310:737-743.

21. Buroker, T. R. et al. (1985) A controlled evaluation of recent approaches to biochemical modulation or enhancement of 5-flourouracil therapy in colorectal carcinoma. *Journal of Clinical Oncology,* 3:1624-1631.

22. Gastrointestinal Tumor Study Group. (1985) Prolongation of the disease-free interval in surgically treated rectal carcinoma. *New England Journal of Medicine,* 312:1465-1472.

23. Gastrointestinal Tumor Study Group. (1992) Radiation therapy and Flourouracil with or without Semustine for the treatment of patients with surgical adjuvant adenocarcinoma of the rectum. *Journal of Clinical Oncology,* 10:549-557.

Rev. 9/97 24. Dose, A.M., Mahood, D., Loprinzi, C.L. (1990). A controlled trial of oral cryotherapy for preventing stomatitis in patients receiving 5-FU plus leucovorin (LV). A NCCTG and Mayo Clinic study. *Proc. ASCO, 9:321.*

Rev. 9/97 25. Kennedy P., Presant, C.A., Blayney, D. (1990). Sandostatin therapy for chemotherapy and radiotherapy related diarrhea. *Proc. ASCO, 9:324.*

Rev. 4/98 **This model informed consent form has been reviewed by the DCT/NCI and is the official consent document for this study. Local IRB changes to this document are allowed. (Sections of this document which are in bold type should always be tried to be used in their entirety.) Editorial changes to these sections may be made as long as they do not change information or intent. If the institutional IRB insists on making deletions or more substantive modifications to the risks or alternatives sections, they may be justified in writing by the investigator and approved by the IRB. Under these circumstances, the revised language and justification must be forwarded to the Eastern Cooperative Oncology Group Coordinating Center for approval before a patient may be registered to this study.**

Rev. 9/97 **Phase III Intergroup Prospectively Randomized Trial of Perioperative 5-FU After Curative Resection, Followed by 5-FU/Leucovorin for Patients with Colon Cancer**

APPENDIX I

Suggested Patient Consent Form

Research Studies

I, ___________________________________, willingly agree to participate in this study which has been explained to me by Dr. _______________________. This research study is being conducted by the Eastern Cooperative Oncology Group and by _____________________________.
(Institution)

Purpose of the Study

Rev. 4/98 It has been explained to you that you have colon cancer. **You have been invited to participate in this research study.** This study involves the use of chemotherapy given immediately after your colon operation
Rev. 4/98 (this type of chemotherapy is also referred to as perioperative chemotherapy). **The purpose of this study is to see if early chemotherapy given into your vein continuously for 7 days after the operation will decrease the chance of the cancer returning.**

Description of Procedures

This study involves the use of chemotherapy given immediately after your colon operation. It is not clear at the present time if receiving a drug called 5-FU immediately after your operation is better than not receiving it. For this reason, the perioperative 5-FU will be offered to you based upon chance using a method of selection called randomization. Randomization means that your physician will call a statistical office which will assign one of the options to you, and that the chances of your receiving either one of the two options offered are approximately equal.

Rev. 4/98 **If you enter this study you will be randomly assigned either to get the 7 days of continuous intravenous 5-fluorouracil (5-FU) or not to receive any perioperative therapy. After surgery, your colon cancer will be classified into Dukes' categories (A, B1, B2, B3, C, or D) by the pathology department at the hospital, and you will be treated as follows:**

1. **If your tumor is in the "early stage" category (A, B1) or the "metastatic" category (D), and you are assigned to receive chemotherapy right after your colon operation, that chemotherapy will be stopped, and you will receive no further chemotherapy. You will also be taken off the study, and your doctor will treat you as he/she sees best.**

   If your tumor is in this stage, and you are <u>not</u> assigned to receive chemotherapy right after your operation, you will also be taken off the study and treated as your doctor sees best.

2. **If your tumor is in the "low risk" category (B2), and you are assigned to receive chemotherapy right after your colon operation, you will receive the full course of perioperative 5-FU. You will then be followed to make sure your cancer does not return, but you will not receive any additional chemotherapy.**

   If your tumor is in this category, and you are <u>not</u> assigned to receive chemotherapy right after your operation, you will also be followed to make sure your cancer does not return, but you will not receive any additional chemotherapy.

   If your cancer does return, you will be taken off the study and treated as your doctor sees best.

3. **If your tumor is in the "moderate risk" category (B3 or C), and you are assigned to receive chemotherapy right after your colon operation, you will receive the full course of perioperative 5-FU. You will also receive standard chemotherapy starting one month after the operation. The standard chemotherapy will consist of one injection of 5-FU in your vein for 5 consecutive days.**

Rev. 9/97 **You will also be receiving leucovorin by IV on the same schedule as the 5-FU. The 5-FU and leucovorin will be given monthly for a total of 6 months (6 cycles).**

If your tumor is in the "moderate risk" category, and you are <u>not</u> assigned to receive chemotherapy right after your operation, you will go on to receive the standard chemotherapy described in the above paragraph.

If your cancer does return, you will be taken off the study and treated as your doctor sees best.

Rev. 9/97 **The only investigational part of this protocol is the administration of chemotherapy during the period right after your colon operation. The operation and the use of 5-FU/leucovorin are all standard treatment.**

Risks and Discomforts

Rev. 4/98 The perioperative 5-FU dose used in this study has been tested and shown to be safe to give after an operation. However, all drugs have side effects. **The perioperative 5-FU used in this program may cause all, some, or none of the side effects listed. In addition, there is always the risk of very uncommon or previously unknown side effects occurring.**

Continuous 5-FU may cause your white blood cell count to go down. This may make you more vulnerable to infections. It may also cause a decrease in your platelet count which could make you more prone to bleeding.

Since the 5-FU will be started within 24 hours of your operation, it may increase the risk of a complication after surgery such as an infection or delayed healing of the colon or abdominal wound. It may cause you to have mouth sores. It also may cause you to have chest pains (angina). You understand that if any of these conditions listed occur, the 5-FU will be stopped immediately.

Rev. 9/97 **If your cancer is more advanced, you will also receive 5-FU and leucovorin one month after the operation.**

Rev. 3/95,9/97, 8/99 **You should expect that the chemotherapy will cause side effects. Specifically, all the drugs may decrease the function of your bone marrow, lowering the white blood count and platelet count. These conditions can increase your susceptibility to infections, bruising, and bleeding. These drugs may also cause nausea, vomiting, fatigue, and diarrhea. In addition, leucovorin may cause skin rash, nausea, upset stomach, diarrhea, hives, itching, wheezing, headache**.

Rev. 9/97

5-FU may cause mouth ulcers, temporary hair loss, sensitivity to light, and staggering gait. Allergic sensitization has been reported in a few cases. The combination of these drugs may cause severe gastrointestinal toxicity. The symptoms may be exhibited as mild to severe mouth ulcers as well as moderate to severe diarrhea. The mouth ulcers may be severe enough to inhibit taking an adequate amount of fluids by mouth (orally), resulting in severe dehydration. It is <u>extremely</u> important that you report all symptoms that you experience to your physician.

Rev. 4/98 Your physician will be checking you closely to see if any of these side effects are occurring. Routine blood and urine tests will be done to monitor effects of treatment. **A chest x-ray will be done every 6 months during years 1 and 2, and barium enema or colonoscopy will be done every 12 months.** Many side effects disappear

after the 5-FU is stopped. In the meantime, your doctor may prescribe medication to keep these side effects under control. You understand that treatment to help control side effects could result in added costs. This institution is not financially responsible for treatments of side effects caused by the study drugs.

Contact Persons

Rev. 4/98 **In the event that physical injury occurs as a result of this research, facilities for treatment of injury [____________ will, will not] be available. You understand, however, that you will not automatically be provided with reimbursement for medical care or other compensation. For more information concerning the research and research-related risks or injuries, you can notify Dr. ____________, the investigator in charge at ________________________ (Telephone). In addition, you may contact ________________________at ____________________________________ (Telephone) for information regarding patients' rights in research studies.**

Alternatives

Perioperative chemotherapy, which is the only investigational part of this protocol, is not routine treatment.
Rev. 9/97, 4/98 **Standard therapy for patients with colon tumors in the "moderate risk" category (B3 or C) is 5-FU/leucovorin starting 3-4 weeks after surgery. There is no evidence that standard therapy alone would work better than what is proposed in this study.** An additional alternative is no further therapy. You understand that your doctor can provide detailed information about your disease and the benefits of the various treatments available. You have been told that you should feel free to discuss your disease and your prognosis with the doctor.

The physician involved in your care will be available to answer any questions you have concerning this program. In addition, you understand that you are free to ask your physician any questions concerning this program that you wish in the future. You will be advised of procedures related solely to research which would not otherwise be necessary. These will be explained to you by your physician. Some of these procedures may result in added costs and some of these costs may not be covered by insurance. Your doctor will discuss these with you.

Benefits

Rev. 4/98 **The benefit from this study is that you may receive a form of chemotherapy (perioperative 5-FU) that is experimental and may be beneficial to you.** If you are not randomized to that treatment, you understand that you will still receive the standard chemotherapy for colon cancer if it is indicated.

Voluntary Participation

Rev. 4/98 **Participation in this study is voluntary. No compensation for participation will be given. You understand that you are free to withdraw your consent to participate in this treatment program at any time without prejudice to your subsequent care. Refusing to participate will result in no penalty or loss of benefits.** You are free to seek care from a physician of your choice at any time. **If you do not take part in or withdraw from the study, you will continue to receive care. In the event that you withdraw from the study, you will continue to be followed and clinical data will continue to be collected from your medical records.**

Cost and Payments

Blood tests, laboratory, and physicians' charges will be billed to you in the same fashion as if you were not part of the study. These tests are felt to be a part of good medical care and are usually covered by most insurance companies.

Use of Tumor Tissue Taken at the Time of Surgery

You understand that tumor and normal surrounding tissue taken at the time of your colon operation may be used for special tests that are part of another laboratory study. In that study, researchers will look at the tissue cells from your tumor to see if there are any special gene "markers." Doctors hope to use the results obtained from the tests done on your tumor to help them predict in the future how patients will do after they are diagnosed with colon cancer and how they will do after chemotherapy.

Rev. 4/98

Confidentiality

Rev. 4/98 **You understand that a record of your progress while on this study will be kept in a confidential form at _________________________________ and also in a computer file at the statistical headquarters**
(Institution)
of the Eastern Cooperative Oncology Group. The confidentiality of the central computer record is carefully guarded. During their required reviews, representatives of the Food and Drug Administration (FDA) and the National Cancer Institute (NCI) and sponsoring agencies may have access to medical records which contain your identity. However, no information by which you can be identified will be released or published. Histopathologic material, including slides, may be sent to a central office for additional review.

* *

I have read all of the above, asked questions, received answers concerning areas I did not understand and I willingly give my consent to participate in this program. Upon signing this form, I will receive a copy.

(Patient Signature)	(Date)
(Witness Signature)	(Date)

Rev. 4/98

Rev. 4/98 **I, _______________________, willingly agree that any tissue collected for this protocol may be stored at the ECOG Pathology Coordinating Office. This remaining tissue may be used for future research that could include genetic research (about diseases that are passed on in families). This research will not have an effect on my care, therefore, neither I nor my doctor will receive results of this testing. No medical report will be added to my records. My medical records may be reviewed in the future for purposes of obtaining more information about my health but my name and address will remain confidential and will not be released. The tissue will be used for research purposes only, it will not**
Rev. 4/98 **be sold and may not have a direct benefit to me or my cancer. Tissue may also be used for additional research as specifically approved by ECOG.**

If I decide now that my tissue can be kept for research, I can change my mind at any time. I just need to contact my doctor and withdraw my consent for the use of my tissue for research.

I have read all of the above, asked questions and received answers concerning areas that I did not understand. I willingly consent to allow my tissue to be stored for future research.

* *

(Patient Signature)	(Date)
(Witness Signature)	(Date)

Rev. 9/97

Phase III Intergroup Prospectively Randomized Trial of Perioperative 5-FU After Curative Resection, Followed by 5-FU/Leucovorin for Patients with Colon Cancer

APPENDIX II

COMMON TOXICITY CRITERIA

		0	1	2	3	4
Leukopenia	WBC x 10^3<br>Granulocytes/Bands<br>Lymphocytes	≥4.0<br>≥2.0<br>≥2.0	3.0 - 3.9<br>1.5 - 1.9<br>1.5 - 1.9	2.0 - 2.9<br>1.0 - 1.4<br>1.0 - 1.4	1.0 - 1.9<br>0.5 - 0.9<br>0.5 - 0.9	<1.0<br><0.5<br><0.5
Thrombocyto-penia	Plt x 10^3	WNL	75.0 - normal	50.0 - 74.9	25.0 - 49.9	<25.0
Anemia	Hgb	WNL	10.0 - normal	8.0 - 10.0	6.5 - 7.9	<6.5
Hemorrhage (Clinical)	- - - - -	none	mild, no transfusion	gross, 1-2 units transfusion/episode	gross, 3-4 units transfusion/episode	massive, >4 units transfusion/episode
*Infection	- - - - -	none	mild, no active Rx	Moderate, localized infection requires active Rx	severe, systemic infection requires active Rx, specify site	life-threatening, sepsis, specify site
Fever in absence of infection	- - - - -	none	37.1° - 38.0° C<br>98.7 °- 100.4° F	38.1 ° - 40.0° C<br>100.5 ° - 104.0° F	>40.0° C (>104.0° F) for less than 24 hours	>40.0° C (104.0° F) for >24 hrs or fever with hypotension
	**• Fever felt to be caused by drug allergy should be coded as allergy.<br>• Fever due to infection is coded under infection only.**					
GU	Creatinine	WNL	< 1.5 x N	1.5 - 3.0 x N	3.1 - 6.0 x N	>6.0 x N
	Proteinuria	No change	1+ or <0.3g% or <3g/l	2-3+ or 0.3 - 1.0g% or 3 - 10g/l	4+ or >1.0g% or >10g/l	nephrotic syndrome
	Hematuria	neg	micro only	gross, no clots	gross + clots	requires transfusion
	*BUN	<1.5 x N	1.5 - 2.5 x N	2.6 - 5 x N	5.1 - 10 x N	>10 x N
	**• Urinary tract infection should be coded under infection, not GU.<br>• Hematuria resulting from thrombocytopenia should be coded under hemorrhage, not GU.**					
GI	Nausea	none	able to eat reasonable intake	intake significantly decreased but can eat	no significant intake	- - - - -
	Vomiting	none	1 episode in 24 hours	2-5 episodes in 24 hours	6-10 episodes in 24 hours	>10 episodes in 24 hrs or requiring parenteral support
	Diarrhea	none	increase of 2-3 stools/day over pre-Rx	increase of 4-6 stools/day, or nocturnal stools, or moderate cramping	increase of 7-9 stools/day or incontinence, or severe cramping	increase of ≥10 stools/day or grossly bloody diarrhea, or need for parenteral support
	Stomatitis	none	painless ulcers, erythema, or mild soreness	painful erythema, edema, or ulcers, but can eat	painful erythema, edema or ulcers, and cannot eat	requires parenteral or enteral support
Liver	Bilirubin	WNL	- - - - -	<1.5 x N	1.5 - 3.0 x N	>3.0 x N
	Transaminase (SGOT, SGPT)	WNL	≤2.5 x N	2.6 - 5.0 x N	5.1 - 20.0 x N	>20.0 x N
	Alk Phos or 5'nucleotidase	WNL	≤2.5 x N	2.6 - 5.0 x N	5.1 - 20.0 x N	>20.0 x N
	Liver - clinical	no change from baseline	- - - - -	- - - - -	precoma	hepatic coma
	**• Viral Hepatitis should be coded as infection rather than liver toxicity.**					
Pulmonary	- - - - -	none or no change	asymptomatic, with abnormality in PFTs	dyspnea on significant exertion	dyspnea at normal level of activity	dyspnea at rest
	**• Pneumonia is considered infection and not graded as pulmonary toxicity unless felt to be resultant from pulmonary changes directly induced by treatment.**					
Cardiac	Cardiac dysrhythmias	none	asymptomatic, transient, requiring no therapy	recurrent or persistent, no therapy required	requires treatment	requires monitoring, or hypotension or ventricular tachycardia or fibrillation
	Cardiac function	none	asymptomatic, decline of resting ejection fraction by less than 20% of baseline value	asymptomatic, decline of resting ejection fraction by more than 20% of baseline value	mild CHF, responsive to therapy	severe or refractory CHF

COMMON TOXICITY CRITERIA

		0	1	2	3	4
	Cardiac-- ischemia	none	non-specific T-wave flattening	asymptomatic, ST and T wave changes suggesting ischemia	angina without evidence for infarction	acute myocardial infarction
	Cardiac-- pericardial	none	asymptomatic effusion, no intervention required	pericarditis (rub, chest pain, ECG changes)	symptomatic effusion; drainage required	tamponade; drainage urgently required

		0	1	2	3	4
Blood Pressure	Hypertension	none or no change	asymptomatic, transient increase by >20 mm Hg (D) or to >150/100 if previously WNL. No treatment required	recurrent or persistent increase by >20 mm Hg (D) or to >150/100 if previously WNL. No treatment required	requires therapy	hypertensive crisis
	Hypotension	none or no change	changes requiring no therapy (including transient orthostatic hypotension)	requires fluid replacement or other therapy but not hospitalization	requires therapy and hospitalization; resolves within 48 hours of stopping the agent	requires therapy and hospitalization for >48 hours after stopping the agent
Skin	- - - - -	none or no change	scattered macular or papular eruption or erythema that is asymptomatic	scattered macular or papular eruption or erythema with pruritus or other associated symptoms	generalized symptomatic macular, papular or vesicular eruption	exfoliative dermatitis or ulcerating dermatitis
Allergy	- - - - -	none	transient rash, drug fever <38° C, 100.4° F	urticaria, drug fever ≥ 38°C, 100.4°F, mild bronchospasm	serum sickness, bronchospasm, requires parenteral meds	anaphylaxis
*Phlebitis		none	arm	thrombophlebitis, leg	hospitalization	embolus
Local		none	pain	pain and swelling, with inflammation or phlebitis	ulceration	plastic surgery indicated
Alopecia	- - - - -	no loss	mild hair loss	pronounced or total hair loss	- - - - -	- - - - -
Weight gain/loss	- - - - -	<5.0%	5.0 - 9.9%	10.0 - 19.9%	≥20%	- - - - -
NEUROLOGIC – Sensory	neuro -- sensory	none or no change	mild paresthesias; loss of deep tendon reflexes	mild or moderate objective sensory loss; moderate paresthesias	severe objective sensory loss or paresthesias that interfere with function	- - - - -
	neuro -- vision	none or no change	- - - - -	- - - - -	symptomatic subtotal loss of vision	blindness
	neuro -- hearing	none or no change	asymptomatic, hearing loss on audiometry only	tinnitus	hearing loss interfering with function but correct-able with hearing aid	deafness, not correctable
Motor	neuro -- motor	none or no change	subjective weakness; no objective findings	mild objective weakness without significant impairment of function	objective weakness with impairment of function	paralysis
	neuro -- constipation	none or no change	mild	moderate	severe	ileus >96 hours
Psych	neuro -- mood	no change	mild anxiety or depression	moderate anxiety or depression	severe anxiety or depression	suicidal ideation
Clinical	neuro -- cortical	none	mild somnolence or agitation	moderate somnolence or agitation	severe somnolence, agitation, confusion, disorientation or hallucinations	coma, seizures, toxic psychosis
	neuro -- cerebellar	none	slight incoordination, dysdiadockinesis	intention tremor, dysmetria, slurred speech, nystagmus	locomotor ataxia	cerebellar necrosis
	neuro -- headache	none	mild	moderate or severe but transient	unrelenting and severe	- - - - -
Metabolic	Hyperglycemia	<116	116 - 160	161 - 250	251 - 500	>500 or ketoacidosis
	Hypoglycemia	>64	55 - 64	40 - 54	30 - 39	<30
	Amylase	WNL	<1.5 x N	1.5 - 2.0 x N	2.1 - 5.0 x N	>5.1 x N
	Hypercalcemia	<10.6	10.6 - 11.5	11.6 - 12.5	12.6 - 13.5	≥13.5
	Hypocalcemia	>8.4	8.4 - 7.8	7.7 - 7.0	6.9 - 6.1	≤6.0
	Hypomagnesemia	>1.4	1.4 - 1.2	1.1 - 0.9	0.8 - 0.6	≤0.5
Coagulation	Fibrinogen	WNL	0.99 - 0.75 x N	0.74 - 0.50 x N	0.49 - 0.25 x N	≤0.24 x N
	Prothrombin time	WNL	1.01 - 1.25 x N	1.26 - 1.50 x N	1.51 - 2.00 x N	>2.00 x N
	Partial thromboplastin time	WNL	1.01 - 1.66 x N	1.67 - 2.33 x N	2.34 - 3.00 x N	>3.00 x N

* denotes ECOG specific criteria

Rev. 9/97

Phase III Intergroup Prospectively Randomized Trial of Perioperative 5-FU After Curative Resection, Followed by 5-FU/Leucovorin for Patients with Colon Cancer

APPENDIX III

Pathology Submission Guidelines

The following items are included in Appendix III:

1. Guidelines for Submission of Pathology Materials (instructional sheet for data managers)
2. Instructional memo to submitting pathologists
3. List of Requested Materials for E1292
4. Patient Information Form

GUIDELINES FOR SUBMISSION OF PATHOLOGY MATERIALS

The following items are needed for the submission of pathology materials for E1292:

- Instructional memorandum to the submitting pathologist
- List of Requested Material
- Patient Information Form

Instructions:

1. Complete the top portion of the Patient Information Form.
2. Forward the pathologist's instructional memo, the List of Requested Material, and the Patient Information Form to the appropriate pathologist.

   The pathologist should return to you the required pathologic samples and reports, along with the completed Patient Information Form.
3. Make sure the pathologist has retained a copy of the Patient Information Form for his/her records.
4. Double check that **ALL** required pathology materials have been submitted (see appropriate List of Requested Material).

Rev. 7/96 ECOG Institutions:

5. Mail paraffin samples and slides to: (Must be submitted within 1 month of surgery)

Rev. 3/95, 9/95, 9/97
ECOG Pathology Coordinating Office
Evanston Hospital- Room B624
2650 Ridge Avenue
Evanston, IL 60201-1797

Pathology specimens submitted for a patient WILL NOT be processed by the Pathology Coordinating Office until all necessary items are received.

Rev. 9/97 **Frozen samples are no longer being collected for this study.**

If you have any questions concerning the above instructions, contact the Pathology Coordinator
Rev. 3/95, 9/97 at the ECOG Pathology Coordinating Office TEL: (847) 570-1133 or FAX: (847) 570-1135.

Rev. 7/96 CALGB Institutions:

The following should be submitted to (Within one month of surgery):

CALGB Pathology Office
Maurice Barcos, M.D., Ph.D.
Roswell Park Cancer Institute
Department of Pathology
Elm and Carlton Streets
Buffalo, NY 14263
Tel: 716-845-4443
Fax: 716-845-8077

1. One paraffln embedded block with REPRESENTATIVE TUMOR properly identifying:

   a. Patient's name
   b. CALGB patient number and ECOG patient number
   c. CALGB study number and ECOG study number

2. Original Patient Information Form (H&E Section to be completed by CALGB Pathology Office)

3. Original CALGB Form C-350

4. A copy of the responsible pathologist's surgical and pathology report from the TREATING institution, and, if applicable, the REFERRING institution.

   These tissue blocks will be sectioned by the CALGB Pathology Office, according to ECOG Pathology Coordinating Office specifications, and then sent to the ECOG PCO, along with the completed Patient Information Form.

MEMORANDUM

TO: ______________________________
Submitting Pathologists

FROM: Stanley Hamilton, M.D.
Chair
ECOG Pathology Committee

Rev. 8/99 **SUBJECT:** ***Submission of Pathology Materials for E1292 (CALBG 9395, INT 0136)***

The patient named on the attached Patient Information Form has been entered onto an ECOG protocol requiring pathology review.

Please complete appropriate sections of the Patient Information Form. Keep a copy for your own records, and return the completed Form, the surgical pathology report(s), the slides and blocks, and any other required material (see attached List of Requested Material) to the Data Manager. The Data Manager will forward all required pathology material to the ECOG Pathology Coordinating Office or Dr. Stanley Hamilton's office at Johns Hopkins.

Rev. 3/95 **Note:** ***Paraffin blocks and slides are being collected from this study for the purpose of tissue banking and will be retained indefinitely at the EOCG Central Tissue Repository for use in future studies. The blocks/slides will be available on specific request for purposes of individual patient management.***

Rev. 3/95 **Note:** ***Frozen tissue is no longer being collected for this study.***

Rev. 9/97

Rev. 3/95, 9/97 If you have any questions concerning the above instructions, contact the Pathology Coordinator at the ECOG Pathology Coordinating Office TEL: (847) 570-1133 or FAX: (847) 570-1135.

Rev. 3/95, 9/97

Thank you.

Rev. 3/95 The ECOG Data Manager at your institution is:

Name: ______________________________

Address: ______________________________

____

Phone: ______________________________

Thank you.

LIST OF REQUESTED MATERIAL

Rev. 9/97

E1292	**Phase III Intergroup Prospectively Randomized Trial of Perioperative 5-FU After Curative Resection, Followed by 5-FU/Leucovorin for Patients with Colon Cancer**

1. Paraffin/Slide Samples (submitted to the ECOG Pathology Coordinating Office)

Rev. 3/95 **NOTE: Submission of paraffin blocks and slides is mandatory for ECOG patients.**

a. Patient Information Form.

b. Surgical pathology report.

c. At least one (1) formalin-fixed, paraffin-embedded block from a representative area of the primary tumor.

d. One (1) formalin-fixed, paraffin-embedded block from normal colonic mucosa (ECOG institutions only).

e. 1 H & E stained slide from each block of tumor or normal colonic mucosa submitted (ECOG institutions only).

(Any extra tumor blocks would be appreciated by Dr. Hamilton and the ECOG Central Tissue Repository).

Rev. 7/96
Rev. 9/97

2. Frozen tissue samples are no longer being collected for this study.

Patient Information Form

Date of Surgery: ____/____/____
mo day year

Patient Name: ____
First MI Last

ECOG Patient Sequence No. for E1292: __________________________

Rev. 7/96 CALGB Patient ID number for CALGB 9395 (CALGB institutions only): ___________________________

Institution: ____

Affiliate (if appropriate): ____

Patient's Hospital Number: ______________________________________

Type of Tissue: (check those that apply)

Type	Number of Blocks/ Slides Submitted	Surgical Pathology ID Number
_ Paraffin Blocks<br>___ Tumor Tissue<br>___ Normal Colonic Mucosa	____________<br>____________	____________<br>____________
___ H & E Stained Slides<br>___ Tumor Tissue<br>___ Normal Colonic Mucosa	____________<br>____________	____________<br>____________

Rev. 9/97

Pathologist Name: ____

FOR OFFICE USE ONLY:
Date Sample Received: ____/____/____
mo day year

Items Received :____

Rev. 9/97

Phase III Intergroup Prospectively Randomized Trial of Perioperative 5-FU After Curative Resection, Followed by 5-FU/Leucovorin for Patients with Colon Cancer

APPENDIX IV

Modified Dukes' Classification

Dukes' A - Invasion into sub-mucosa but not into muscular wall, and no regional lymph node involvement.

Dukes' B_1 - Invasion into but not through the muscular wall, and no regional lymph node involvement.

Dukes' B_2 - Transmural penetration of muscular wall with tumor involvement into or through the serosa, but no regional lymph node involvement.

Dukes' B_3 - Tumor invading directly into other organs or tissues, but no regional lymph node involvement.

Dukes' C - Tumor invading to any depth with involvement of the regional lymph nodes.

Dukes' D - The tumor has extended beyond the scope of curative operation resection, either on the basis of distant metastasis or unresectable local regional disease. Patients with isolated, distant, or non-contiguous intra-abdominal metastases, even if resected, are considered to have Dukes' D tumors.

Rev. 9/97

Phase III Intergroup Prospectively Randomized Trial of Perioperative 5-FU After Curative Resection, Followed by 5-FU/Leucovorin for Patients with Colon Cancer

APPENDIX V

Technique of Colon Resection

The following guidelines will be implemented when carrying out the operation procedure.

1. Instillation of chemicals into the lumen of the colon is prohibited. Saline irrigation is permitted if so desired.

2. Mobilization of the mesentery is recommended but is optional.

3. The margins of the resected specimen must be free of tumor.

4. The segment of colon removed must be accompanied by the attached resected mesentery.

5. It is recommended that the following vessels be ligated:

   a. For lesions of the cecum and ascending colon: the right colic artery is ligated.

   b. For lesions of the hepatic flexure: the right branch of the middle colic artery as well as the right colic artery.

   c. For lesions of the transverse colon: the middle colic artery.

   d. For lesions of the descending colon: the left colic artery is ligated.

   e. For lesions of the sigmoid: the sigmoidal branch of the inferior mesenteric artery is ligated.

   f. For lesions located in the transverse colon or at the hepatic or splenic flexures: the greater omentum should be resected.

6. It is recommended that the spleen not be removed when there is a lesion in the splenic flexure unless necessary to achieve histologically free margins.

7. If the tumor invades the viscera or abdominal wall (as long as it is contiguous with the main tumor) the adjacent structure should be removed in continuity. If direct extension of tumor into an ovary occurs, removal of the ovary is permitted.

8. Patients with obstructing carcinoma may have a pre-resection decompression; definitive resection should take place.

9. The type of suture material employed in the performance of the anastomosis is optional but must be recorded.

Phase III Intergroup Prospectively Randomized Trial of Perioperative 5-FU After Curative Resection, Followed by 5-FU/Leucovorin for Patients with Colon Cancer

APPENDIX VI

Expanded Participation Project (EPP) Instructions

1.0 EPP Randomization and Registration Procedures

I. EPP institutions will register a patient on-line through the Clinical Trials Management Unit (CTMU) either pre-operatively within 2 weeks or post-operatively within 24 hours of surgery. Questions pertaining to eligibility criteria should be directed to the CTMU, medical questions should be directed to the Study Chair.

II. A signed HHS 310 form documenting the Institutional Review Board (IRB) approval for this study must be on file at the CTMU before the EPP institution can enter a patient. IRB approval date must be less than one year prior to the date of registration.

III. Once eligibility is confirmed, the CTMU will contact ECOG to randomize the patient. The CTMU will notify the institution by an email upon successful enrollment with ECOG. In addition ECOG will forward confirmation of randomization and treatment assignment to the CTMU for routing to the participating institutions. Please check for errors, and submit any corrections on-line to the CTMU.

2.0 EPP Data Submission

Data must be submitted electronically directly to the CTMU according to the following schedule:

FORM	TIME OF SUBMISSION
**Segment 1***	
E1292 Eligibility Checklist	At registration
ECOG Colon Surgical Adjuvant On-Study form (#559)**<br>*(Surgical pathology and Operative reports***)*	Within 1 week of registration
EPP Toxicity Form	At the end of segment 1(prior to registering to Segment 2)

**Segment 2***	
EPP Toxicity Form	Months 1, 2, 3, and every three months while on protocol therapy
EPP Follow-up Form**	Every 3 months while on protocol treatment and every 6 months after completion of protocol treatment until death
EPP Recurrence Form	At the time of recurrence
EPP Chemotherapy/Immunotherapy/Hormonal Therapy Form	At the completion of protocol therapy
EPP Off-Treatment Form	At the completion of all protocol therapy
EPP Notice of Secondary Malignancy Form	Within 10 days of diagnosis
EPP Death Form	Within 7 days of knowledge of event

* This protocol consists of two segments:
Segment #1: Randomization to peri-operative 5-FU vs. Observation
Segment #2: Registration to observation or adjuvant chemotherapy

** These forms are to be submitted for all canceled patients according to the above schedule.

*** Fax a copy of the Surgical Pathology and Operative Reports to the CTMU ATTN: EPP Protocol Monitor 301-299-3991.

3.0 Pathology Submission

EPP institutions will not be required to submit tissue samples.

4.0 EPP Adverse Drug Reaction

For EPP Institutions, all ADR's are to be faxed to the CTMU (Attn: EPP Protocol Monitor 301-299-3991) using the Adverse Reaction (ADR) Form for Investigational Drugs. These reports will be reviewed and directed to the Eastern Cooperative Oncology Group and appropriate regulatory offices.
Rev. 3/10 ADR reporting is based on the revised NCI Common Toxicity Criteria (version 4.0).

ADRs will be faxed to the CTMU based on the following adverse event reporting requirements table:

**AGENTS**	**1**	**2**	**3**	**4**	**5**
Commercial Agents Expected AE	-----	-----	-----	-----	-----
Commercial Agents Unexpected AE	-----	-----	-----	ADR	ADR

ADR = Adverse Drug Reaction Report (within 7 days)

ADR reports should be submitted via fax within 24 hours of the event. These will be forwarded to the NCI and the Coordinating Group within 5 working days. All ADRs should be reported to the local IRB.

For commercially available drugs, written reporting of any increased incidence of a known ADR is also required in addition to grade 4 and 5 toxicities.

All toxicities, including those with separate reporting requirements described above, must be reported on the Toxicity Form. Deaths are required to be reported via the Death Form within 7 days of knowledge of the event.

5.0 EPP Secondary Malignancy Reporting

Investigators are required to report secondary malignancies occurring on or following treatment on NCI-sponsored protocols using commercial drugs. Reporting of cases of secondary AML/MDS is to be performed using the NCI/CTEP Secondary AML/MDS Report Form. This form should be used in place of DCT Adverse Reaction (ADR) Form for reporting this toxicity. All other secondary malignancies should be reported using the form DCT Adverse Reaction Form. The EPP Notice of Secondary Malignancy must also be completed for all cases of secondary malignancy.
